# Exclusively Breastfed Infant Microbiota Develops over Time and Is Associated with Human Milk Oligosaccharide Intakes

**DOI:** 10.3390/ijms23052804

**Published:** 2022-03-03

**Authors:** Ali Sadiq Cheema, Michelle Louise Trevenen, Berwin Ashoka Turlach, Annalee June Furst, Ana Sophia Roman, Lars Bode, Zoya Gridneva, Ching Tat Lai, Lisa Faye Stinson, Matthew Scott Payne, Donna Tracy Geddes

**Affiliations:** 1 School of Molecular Sciences, The University of Western Australia, Crawley, WA 6009, Australia; alisadiq.cheema@research.uwa.edu.au (A.S.C.); zoya.gridneva@uwa.edu.au (Z.G.); ching-tat.lai@uwa.edu.au (C.T.L.); lisa.stinson@uwa.edu.au (L.F.S.); 2 Centre for Applied Statistics, The University of Western Australia, Crawley, WA 6009, Australia; michelle.trevenen@uwa.edu.au (M.L.T.); berwin.turlach@uwa.edu.au (B.A.T.); 3 Larsson-Rosenquist Foundation Mother-Milk-Infant Center of Research Excellence, University of California San Diego, La Jolla, CA 92093, USA; aloeffler@health.ucsd.edu (A.J.F.); acroman@sdsu.edu (A.S.R.); lbode@health.ucsd.edu (L.B.); 4 Department of Pediatrics, University of California San Diego, La Jolla, CA 92093, USA; 5 Division of Obstetrics and Gynaecology, School of Medicine, The University of Western Australia, Subiaco, WA 6008, Australia; matthew.payne@uwa.edu.au; 6 Women and Infants Research Foundation, Subiaco, WA 6008, Australia

**Keywords:** maternal faecal, human milk, human milk oligosaccharides, human milk bacteria, infant oral, infant faecal, microbiome, 16S rRNA gene, breastfeeding, body composition, intake, concentration, lactation

## Abstract

Temporal development of maternal and infant microbiomes during early life impacts short- and long-term infant health. This study aimed to characterize bacterial dynamics within maternal faecal, human milk (HM), infant oral, and infant faecal samples during the exclusive breastfeeding period and to document associations between human milk oligosaccharide (HMO) intakes and infant oral and faecal bacterial profiles. Maternal and infant samples (*n* = 10) were collected at 2–5, 30, 60, 90 and 120 days postpartum and the full-length 16S ribosomal RNA (rRNA) gene was sequenced. Nineteen HMOs were quantitated using high-performance liquid chromatography. Bacterial profiles were unique to each sample type and changed significantly over time, with a large degree of intra- and inter-individual variation in all sample types. Beta diversity was stable over time within infant faecal, maternal faecal and HM samples, however, the infant oral microbiota at day 2–5 significantly differed from all other time points (all *p* < 0.02). HMO concentrations and intakes significantly differed over time, and HMO intakes showed differential associations with taxa observed in infant oral and faecal samples. The direct clinical relevance of this, however, is unknown. Regardless, future studies should account for intakes of HMOs when modelling the impact of HM on infant growth, as it may have implications for infant microbiota development.

## 1. Introduction

The maternal gut and human milk (HM) microbiota contribute to bacterial colonization of the infant gut, which in turn influences both short- and long-term infant health and development [1,2]. Aberrations to the early-life gut microbiota have been linked to various disorders, such as obesity [3], type 1 diabetes [4], allergies [5], asthma [6], and neurological diseases [7]. Early nutrition is a key factor in directing the composition and function of the infant gut microbiome [8], with breastfeeding being the most significant factor associated with infant gut bacterial structure and function in early life [9]. Therefore, it is important to document the temporal assembly of maternal and infant bacterial communities in the early postnatal period to better understand the foundations for life-long health.

The infant gut microbiome undergoes temporal compositional changes during early life and by 5 years of age is still developing, having not assumed diversity and composition similar to the adult gut [10]. During the first week of life, the infant gut bacterial profile is mainly composed of facultative anaerobes and obligate anaerobes, which are eventually replaced by strict anaerobes as the gut environment shifts in oxygenation [11,12,13,14,15]. HM consumption shifts the infant gut microbiome to a state dominated by *Bifidobacterium* sp. and lactic acid bacteria [16]. The commencement of solid foods again alters the gut microbiome, introducing typical adult gut genera such as *Bacteroides*, *Prevotella*, *Ruminococcus*, and *Clostridium* [16,17]. Although previous studies have characterized the development of the early-life gut microbiota, to date, no such study has been performed in an Australian cohort. This is important, as Australia is a geographically isolated continent, and both infant and adult microbiomes have been shown to vary geographically [18,19,20,21].

In addition to the infant gut, the development of the infant oral cavity is of interest, due to its contribution to oral health and its potential contribution to both the HM and infant gut microbiomes [13,22,23,24]. The acquisition of certain early oral microbiome bacteria such as *Streptococcus mutans* and *Veillonella* sp. has been associated with the development of periodontitis and dental caries [25,26]. During the first three months of life, certain taxa including *Streptococcus mitis*, *Rothia mucilaginosa*, *Veillonella parvula*, *Streptococcus salivarius*, *Gemella haemolysans* and *Veillonella* HB016 dominate the infant oral microbiota [24,27,28]. Changes in bacterial composition within the oral community are associated with feeding methods [28,29], tooth eruption [30] and introduction of solids [28]. Further, bacterial richness and diversity increase over the first 7 years of life [27,28,31]. However, a clear outline of the development of the infant oral microbiota during the exclusive breastfeeding period is still not well-documented.

The maternal microbiome is the primary donor of bacteria to the infant microbiome [13,32,33]. Studies have demonstrated vertical transmission of specific bacterial strains from the maternal to the infant gut [12,13,34,35,36,37], including *Bacteroides* spp., *Bifidobacterium* spp. and *Escherichia coli*. Despite the importance of the maternal gut microbiome as a contributor to the infant gut microbiome, changes to the maternal gut microbiome during the early postnatal period have not been characterized. The maternal gut microbiome undergoes profound changes from the first to third trimester with increased abundance of Proteobacteria and Actinobacteria, decreased richness and increased beta diversity [38]. At one month postpartum, the gut microbiota is similar to that present in the third trimester [38]. One study, which was restricted in taxonomic depth, reported no effect of time on maternal gut bacterial community composition or diversity in the first six months postpartum [39], which suggests that the maternal gut microbiome does not revert to a pre-pregnancy state or change substantially during this time. Given the dramatic remodeling of the maternal gut microbiome during pregnancy, further work is needed to examine the postnatal trajectory of the maternal gut microbiome, particularly in the context of lactation, a time when maternal hormones are in an altered state [40].

In addition to the maternal gut, HM has been highlighted as a source of bacteria for the infant gut microbiome, with evidence of vertical transmission of bacteria to the breastfed infant, particularly *Bifidobacterium* spp. [34,35,36,37]. Two to eighteen bacterial taxa have been reported to form the core HM community [14,18,41,42,43], with *Staphylococcus* sp. and *Streptococcus* sp. typically dominating profiles. Lactation stage, particularly the transition from colostrum to mature milk has been associated with a change in bacterial composition [14,19,44,45]. However, some studies report relatively constant HM bacterial profiles over the first six months postpartum [24,41,46,47,48], with the exception of less abundant genera including *Veillonella* sp., *Leptotrichia* sp., *Prevotella* sp. and *Granulicatella* sp., which tend to shift over time [46,47]. While efforts have been made to characterize the HM microbiome, most of the previous studies are disadvantaged by a major confounder, which is the inclusion of infants fed with complementary foods and/or formula [24,42,46,48]. Therefore, studies focusing on exclusively breastfeeding dyads are required to negate the influence of formula and truly characterize the longitudinal development and/or stability of the HM microbiome during the exclusive breastfeeding period.

While a small number of bacterial taxa are vertically transmitted from mother to infant via HM [34,35,36,37], other HM components are also likely to contribute to the development of the infant gut microbiome. In particular, HM oligosaccharides (HMOs) are of great interest due to their potential to shape HM and infant oral/gut bacterial profiles [49]. HMOs are prebiotic agents that stimulate the growth of specific bacteria, such as *Bifidobacterium* spp., which are the dominant taxa in the breastfed infant gut [50,51]. Additionally, *Bacteroides* sp. and *Streptococcus* sp. have been shown to be able to metabolize HMOs [52,53]. Both positive and negative correlations between HMO concentrations and the relative abundance of certain gut bacteria, including *Bifidobacterium* sp. and *Bacteroides* sp., have been reported [53,54,55,56,57]; however, no previous study has examined the role of HMO daily intakes in shaping the infant oral and gut microbiome. To understand the influence of HMOs on the development of the infant microbiota, an integrated longitudinal analysis of HMO intakes and microbiota composition is needed.

Therefore, the aims of this longitudinal cohort study were to characterize the temporal development of the maternal faecal, HM, infant oral and infant faecal microbiomes over the first four months of life, to track the longitudinal variability of HMOs over this same time period and to determine associations between daily intakes of HMOs and infant oral and faecal bacterial profiles.

## 2. Results

### 2.1. Participant Characteristics

All mothers participating in the current study were Caucasian, had delivered vaginally, had not taken antibiotics, and were exclusively breastfeeding at all time points. Demographics of the 10 mother–infant dyads are shown in Table 1.

### 2.2. PacBio HiFi Sequencing Metrics

The average number of circular consensus sequence (CCS passes) for two SMRT cells was 26, and the minimum predicted sequencing accuracy was 99%, meaning that our estimated sequencing error rate was less than 1%.

### 2.3. Temporal Development of Maternal Faecal Bacterial Profiles

**Figure 1:**
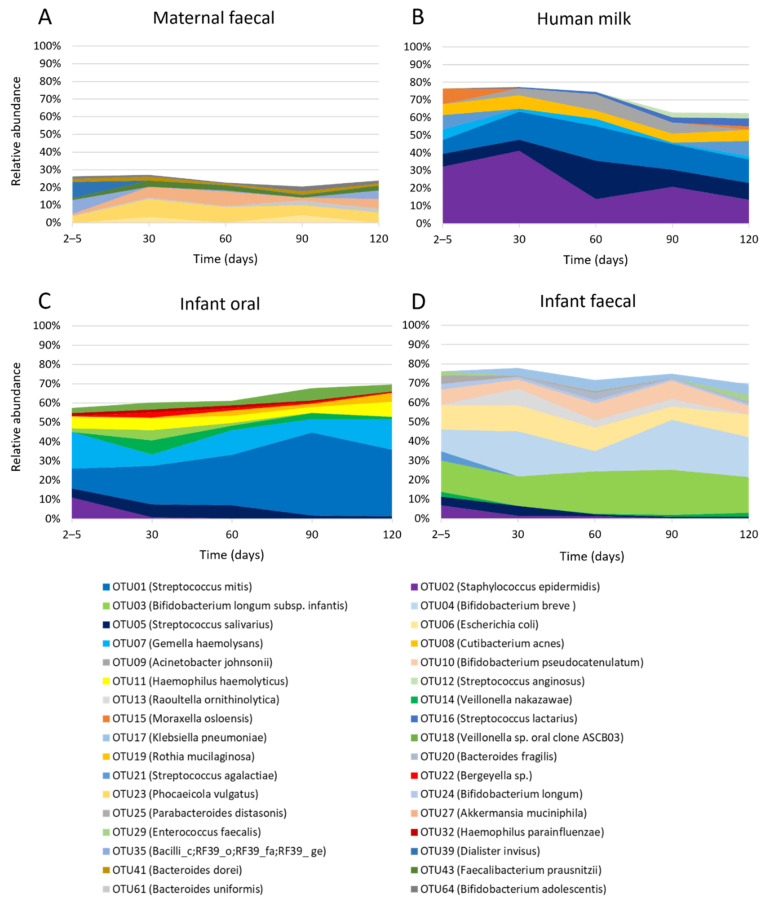
The relative abundance of OTUs constituting ≥ 1% within each sample type. (**A**) Maternal faecal, (**B**) Human milk, (**C**) Infant oral and (**D**) Infant faecal samples.

Within maternal faecal samples, nine OTUs were present at an average relative abundance of >1% (Figure 1A) and these collectively represented 20.4–27.1% of the total bacterial profile. However, the relative abundance of these nine OTUs varied over time, with day 2–5 presenting a different bacterial profile compared to all other time points. The day 2–5 bacterial profile was dominated with OTU39 (*Dialister invisus*) (9.2%) and OTU35 (Bacilli_c;RF39_o;RF39_fa;RF39_ge) (7.8%), while day 30, 60 and 120 profiles were dominated with OTU23 (*Phocaeicola vulgatus*) and OTU27 (*Akkermansia muciniphila*) and day 90 dominated with OTU23 (*P. vulgatus*) and OTU06 (*Escherichia coli*).

**Figure 2:**
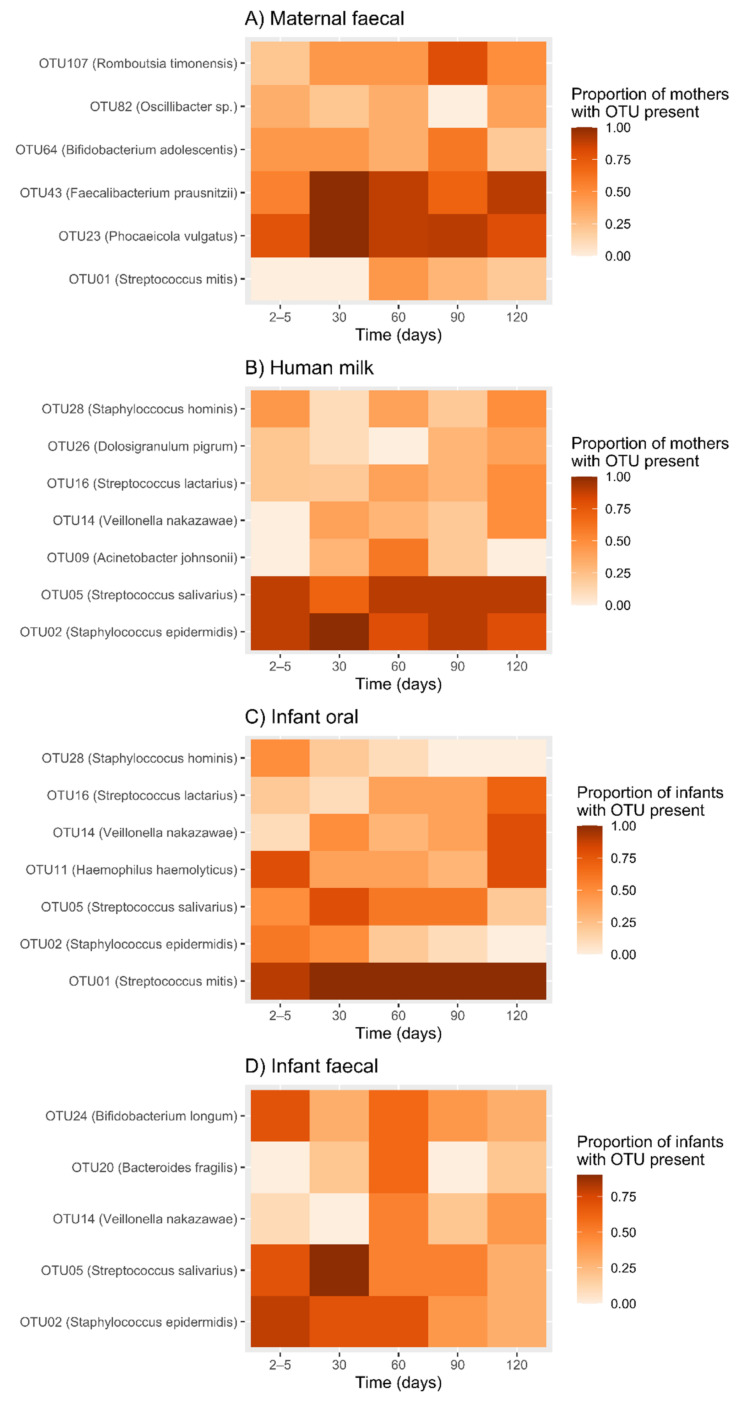
The proportion of mothers and infants at each time point for which an OTU was present. (**A**) Maternal faecal, (**B**) Human milk, (**C**) Infant oral and (**D**) Infant faecal samples. The darkest red/brown colour represents a proportion of 1, indicating that all mothers/infants had that OTU present at that time point. A white shaded box represents a proportion of 0, indicating that no mothers/infants had that OTU present at that time point.

The prevalence of six OTUs (OTU01 (*S. mitis*), OTU23 (*P. vulgatus*), OTU43 (*Faecalibacterium prausnitzii*), OTU64 (*Bifidobacterium adolescentis*), OTU82 (*Oscillibacter* sp.) and OTU107 (*Romboutsia timonensis*)) changed significantly over time (Figure 2A, Table A3).

**Figure A1:**
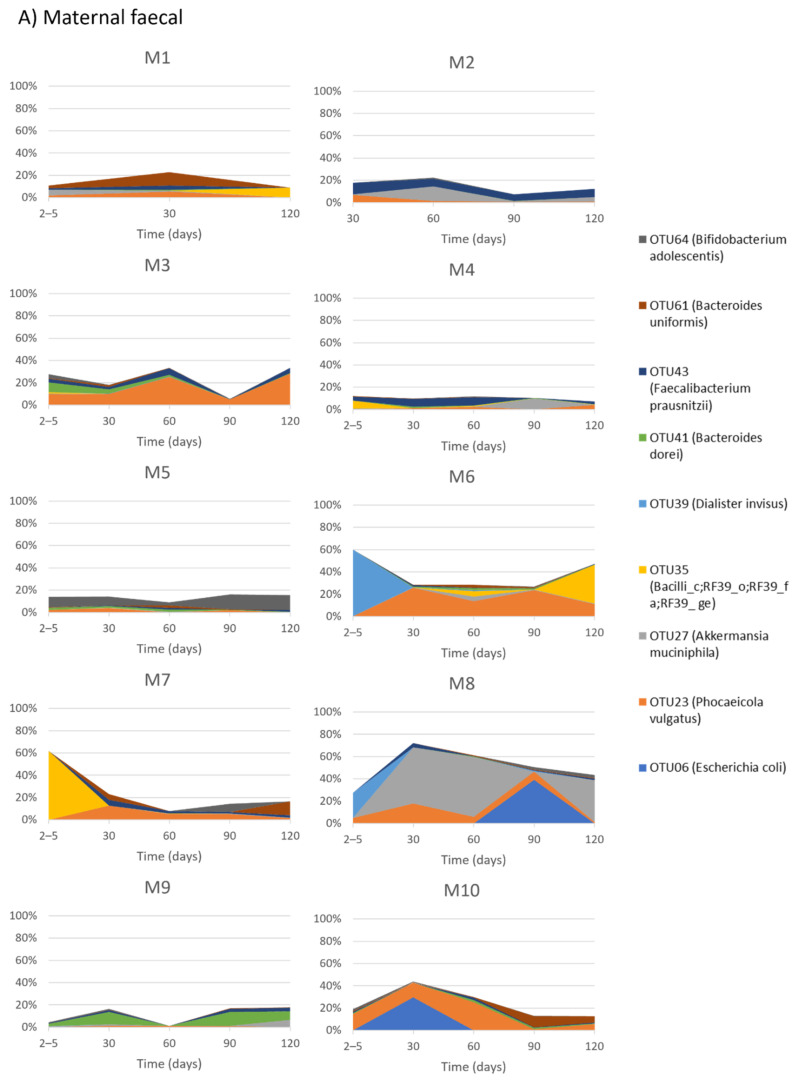

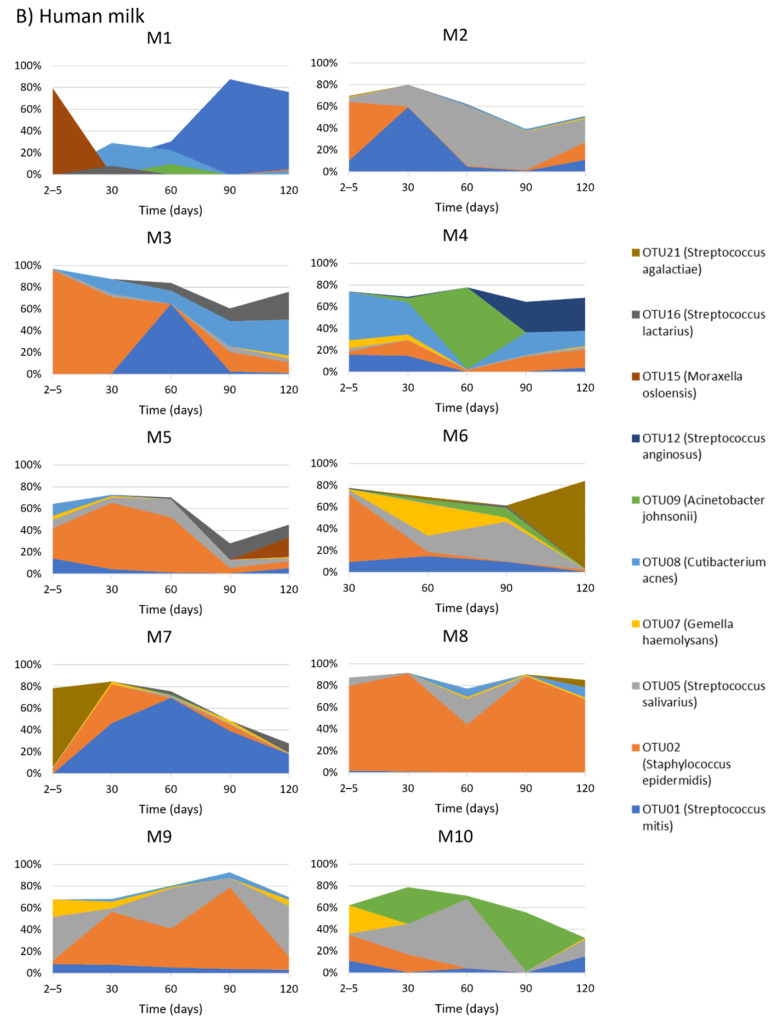

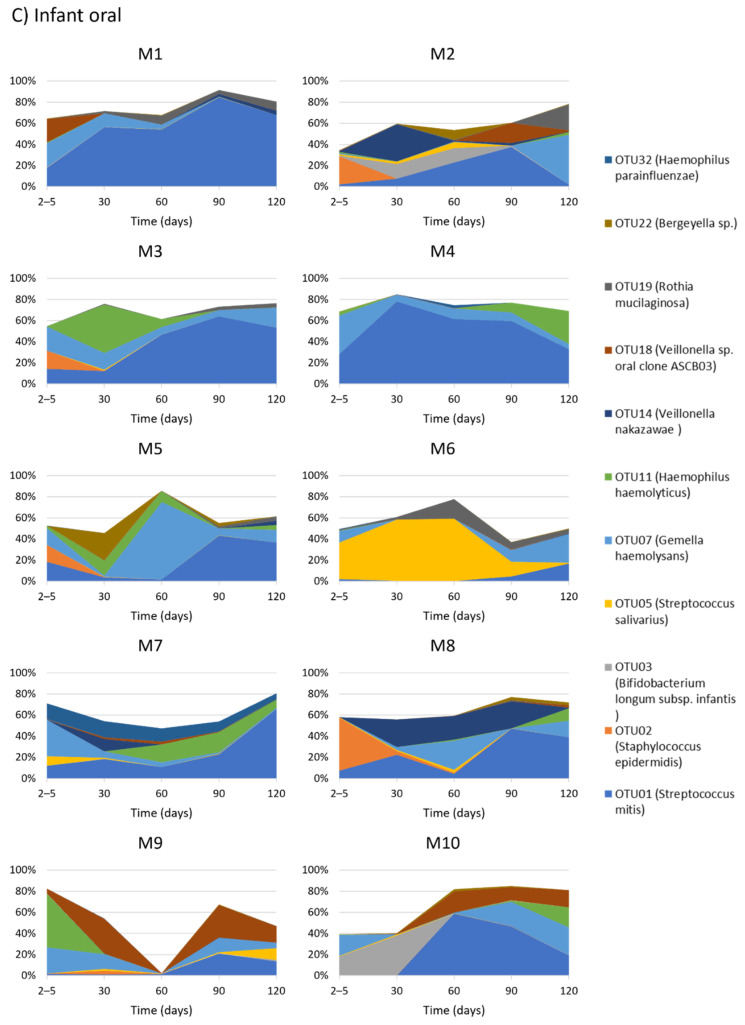

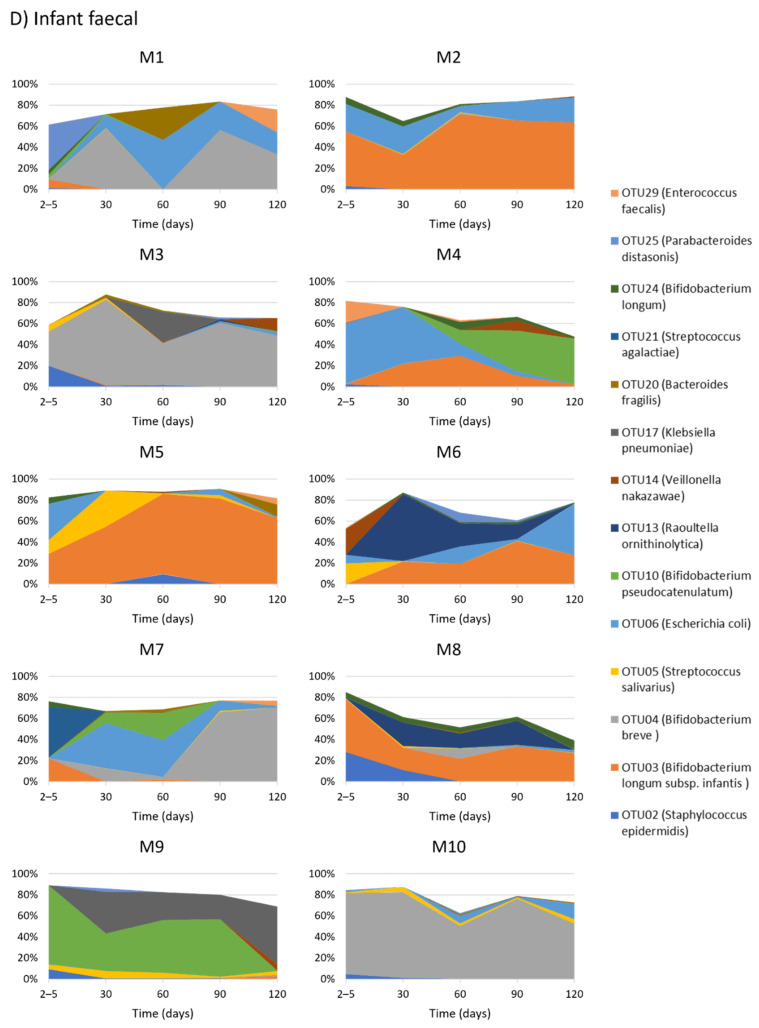
Individual variation in microbiome composition over time. (**A**) Maternal faecal, (**B**) Human milk, (**C**) Infant oral and (**D**) Infant faecal samples. Relative abundance is displayed on the y-axis. Missing maternal faecal samples: M1 days 30 and 60, M2 day 2–5. Missing HM sample: M6 day 2–5.

Additionally, we observed a high level of inter-individual variability in OTU composition, with different taxa dominating different mother’s bacterial profiles at different time points (Figure A1A). We also observed a high level of intra-individual changes in composition over time (Figure A1A).

**Figure 3:**
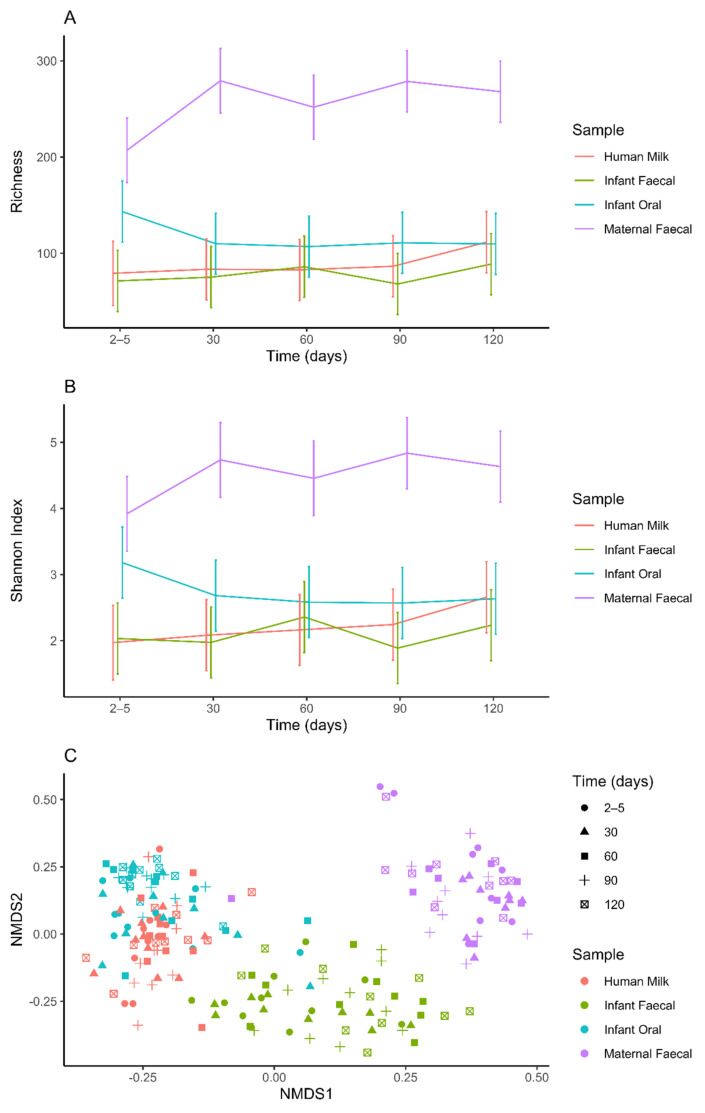
Alpha and beta diversities differ within and between sample types (human milk, infant faecal, infant oral and maternal faecal). (**A**) Richness (number of observed OTUs). (**B**) Shannon diversity. (**C**) NMDS plot of Bray Curtis dissimilarity distances.

Maternal faecal samples were significantly less rich at day 2–5 compared to days 30 (*p* = 0.0023), 90 (*p* = 0.0019) and 120 (*p* = 0.0080) (Figure 3A, Table 2). A significantly lower level of Shannon diversity was also observed at day 2–5 compared to days 30 (*p* = 0.0435) and 90 (*p* = 0.0199) (Figure 3B, Table 2). However, beta diversity within maternal faecal samples was largely stable over time, apart from the day 2–5 sample which was significantly different from the day 30 sample (*p* = 0.0365) (Figure 3C, Table 3).

### 2.4. Temporal Development of Human Milk Bacterial Profiles

Within HM samples, 10 OTUs were present at an average relative abundance of >1% (Figure 1B) and collectively comprised 62.7–77.5% of the total bacterial profile. However, the relative abundance of these 10 OTUs changed over time. The most abundant OTU was OTU02 (*Staphylococcus epidermidis*) which dominated the HM taxa in the first month of life (32.2% at day 2–5, 41.2% at day 30) but fell in relative abundance at the later time points (13.9% at day 60, 20.7% at day 90, and 13.5% at day 120). The relative abundance of the other nine most abundant OTUs also changed over time.

The prevalence of seven OTUs (OTU02 (*S. epidermidis*), OTU05 (*S. salivarius*), OTU09 (*Acinetobacter johnsonii*), OTU14 (*Veillonella nakazawae*), OTU16 (*Streptococcus lactarius*), OTU26 (*Dolosigranulum pigrum*) and OTU28 (*Staphylococcus hominis*)) changed significantly over time (Figure 2B, Table A3).

Similar to the other sample types, we observed a high level of intra-individual and inter-individual variability in OTU composition over time (Figure A1B). Within and between mothers, the relative abundance of bacterial taxa changed across different time points.

Neither richness nor Shannon diversity differed over time within HM samples (Figure 3A,B, Table 2). However, beta diversity in day 30 samples differed significantly from day 60 samples (*p* = 0.0289) (Figure 3C, Table 3).

### 2.5. Temporal Development of Infant Oral Bacterial Profiles

Within infant oral samples, 11 OTUs were present at an average relative abundance of >1% (Figure 1C) and collectively made up 57.5–69.6% of the total bacterial profile. Variation in the relative abundance of these 11 OTUs was observed over time. For example, OTU01 (*S. mitis*), which dominated oral bacterial profiles, increased in relative abundance from day 2–5 (10.4%) to day 30 (20.0%), 60 (26.3%), 90 (43.1%) and 120 (34.6%). Other early colonizers disappeared over time, such as OTU02 (*S. epidermidis*) which was present at day 2–5 (11.1%) but quickly disappeared (<1% relative abundance by day 30 and not present by day 120).

Within individuals the prevalence of seven OTUs (OTU01 (*S. mitis*), OTU02 (*S. epidermidis*), OTU05 (*S. salivarius*), OTU11 (*Haemophilus haemolyticus*), OTU14 (*V. nakazawae*), OTU16 (*S. lactarius*) and OTU28 (*S. hominis*)) changed significantly over time (Figure 2C, Table A3).

There was a large degree of variability of OTUs within infant oral samples (Figure A1C). Additionally, when bacterial taxa composition was compared between infant oral samples, the relative abundance of dominating bacterial taxa changed (Figure A1C).

Neither richness nor Shannon diversity differed over time in infant oral samples (Figure 3A,B, Table 2). However, beta diversity was significantly different at day 2–5 compared to all other time points (all *p* < 0.02). Day 30 samples also differed significantly from day 90 (*p* = 0.0081) and 120 (*p* = 0.0415) samples (Figure 3C, Table 3).

### 2.6. Temporal Development of Infant Faecal Bacterial Profiles

Within infant faecal samples, 14 OTUs were present at an average relative abundance of >1% (Figure 1D), and these collectively made up 69.4–77.9% of the total bacterial profile and varied in relative abundance over time. Overall, four OTUs (OTU03, OTU04, OTU10 and OTU24) mapping to *Bifidobacterium* species dominated the bacterial profile across all time points, increasing from day 2–5 (38.0%) to 90 (59.4%) and then decreasing at day 120 (44.8%).

The presence/absence of five OTUs (OTU02 (*S. epidermidis*), OTU05 (*S. salivarius*), OTU14 (*V. nakazawae*), OTU20 (*Bacteroides fragilis*) and OTU24 (*B. longum*) changed significantly over time (Figure 2C, Table A3).

There was a large degree of intra-individual variation in bacterial composition over time (Figure A1D). Additionally, when bacterial taxa composition was compared between infants, the relative abundance of dominating bacterial taxa differed (Figure A1D).

Neither richness, Shannon diversity (Figure 3A,B, Table 2) nor beta diversity differed over time in infant faecal samples (all *p* > 0.05) (Figure 3C, Table 3).

### 2.7. Alpha and Beta Diversity between Sample Types

Maternal faecal samples were the most rich (all *p* < 0.001) and diverse (Shannon diversity, all *p* < 0.001) at all time points compared to the other sample types (Figure 3A,B, Table 4), except for infant oral versus maternal faecal samples at day 2–5 (*p* = 0.06). Infant oral samples were richer (*p* < 0.005) and more diverse in terms of Shannon diversity (*p* < 0.05) than HM and infant faecal samples at day 2–5. Further, all sample types clustered separately from one another, demonstrating significant dissimilarity in their community structure (Bray–Curtis dissimilarity, all *p* < 0.02), except for HM and infant oral samples at day 60 (*p* = 0.0563) (Figure 3C, Table 5).

### 2.8. HMO Concentrations and Intakes over the First Four Months of Lactation

**Figure 4:**
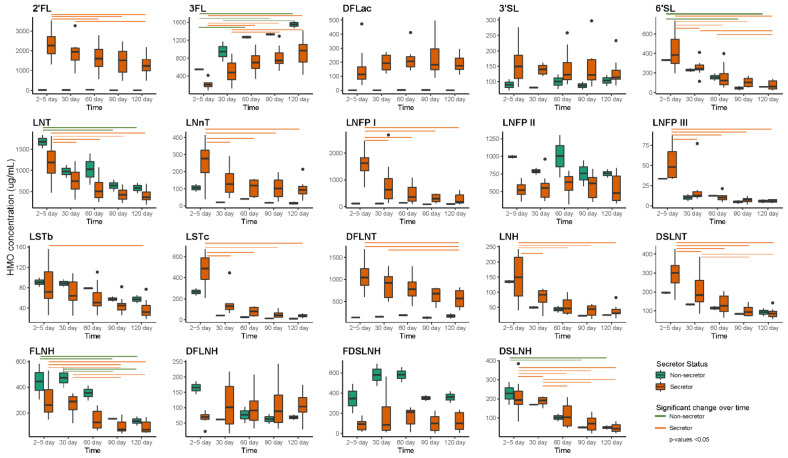
HMO concentrations over time within infants born to mothers with secretor (*n* = 8) and non-secretor (*n* = 2) status. Green and orange lines present significant difference within HMO concentration between time points. 2′FL—2′-fucosyllactose; 3′SL—3′-sialyllactose; 3FL—3-fucosyllactose; 6′SL—6′-sialyllactose; DFLac—difucosyllactose; DFLNH—difucosyllacto-N-hexaose; DFLNT—difucosyllacto-N-tetrose; DSLNH—disialyllacto-N-hexaose; DSLNT—disialyllacto-N-tetraose; FDSLNH—fucodisialyllacto-N-hexaose; FLNH—fucosyllacto-N-hexaose; LNFP I—lacto-N-fucopentaose; LNFP II—lacto-N-fucopentaose II; LNFP III—lacto-N-fucopentaose; LNH—lacto-N-hexaose; LNnT—lacto-N-neotetraose; LNT—lacto-N-tetrose; LSTb—sialyl-lacto-N-tetraose b; LSTc—sialyl-lacto-N-tetraose c.

Six HMOs (2′-fucosyllactose (2′FL), 3-fucosyllactose (3FL), difucosyllacto-N-tetrose (DFLNT), lacto-N-fucopentaose I (LNFP I), lacto-N-fucopentaose II (LNFP II) and lacto-N-tetrose (LNT)) made up the majority of HMO profiles (Figure 4). Concentrations of HMOs varied over time based on maternal secretor status. Among secretor mothers (*n* = 8), the concentrations of 2′FL, 6′-sialyllactose (6′SL), LNT, lacto-N-neotetraose (LNnT), LNFP I, lacto-N-fucopentaose III (LNFP III), sialyl-lacto-N-tetraose b (LSTb), sialyl-lacto-N-tetraose c (LSTc), DFLNT, lacto-N-hexaose (LHN), disialyllacto-N-tetraose (DSLNT), fucosyllacto-N-hexaose (FLNH) and disialyllacto-N-hexaose (DSLNH) decreased significantly from day 2–5 to 120, while 3FL increased over time. Among non-secretor mothers (*n* = 2), 6′SL, LNT, FLNH and DSLNH decreased, while 3FL significantly increased over time.

**Figure 5:**
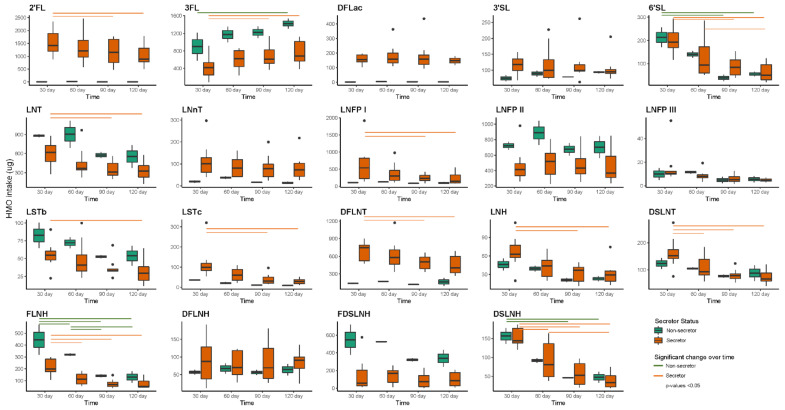
Twenty-four-hour intakes of the HMOs within infants born to mothers with secretor (*n* = 8) and non-secretor (*n* = 2) status. Green and orange lines present significant difference within HMO intakes between time points. 2′FL—2′-fucosyllactose; 3′SL—3′-sialyllactose; 3FL—3-fucosyllactose; 6′SL—6′-sialyllactose; DFLac—difucosyllactose; DFLNH—difucosyllacto-N-hexaose; DFLNT—difucosyllacto-N-tetrose; DSLNH—disialyllacto-N-hexaose; DSLNT—disialyllacto-N-tetraose; FDSLNH—fucodisialyllacto-N-hexaose; FLNH—fucosyllacto-N-hexaose; LNFP I—lacto-N-fucopentaose; LNFP II—lacto-N-fucopentaose II; LNFP III—lacto-N-fucopentaose; LNH—lacto-N-hexaose; LNnT—lacto-N-neotetraose; LNT—lacto-N-tetrose; LSTb—sialyl-lacto-N-tetraose b; LSTc—sialyl-lacto-N-tetraose c.

Additionally, daily intakes of HMOs differed over time in infants born to secretor and non-secretor mothers (Figure 5). Infants born to secretor mothers had higher intakes of 3FL at day 120 compared to day 30, while lower intakes of 2′FL, 6′SL LNT, LNFP I, LSTb, LSTc, DFLNT, LNH, DSLNT, FLNH and DSLNH were observed at day 120 compared to day 30. Infants born to non-secretor mothers had lower intakes of 3FL and higher intakes of 6′SL, FLNH and DSLNH at day 30 compared to day 120. 

### 2.9. Associations between HMO Intake and Infant Oral Microbiota

Intakes of individual HMOs showed both positive and negative associations with different categories of relative abundance and different bacterial OTUs in the infant oral cavity at different time points (Table A4).

At day 30, higher intakes of seven individual HMOs (LNnT, LNFP I, LNFP III, LSTc, LNH, DSLNT, FLNH) were positively associated with different relative abundances of four bacterial OTUs (OTU05 (*S. salivarius*), OTU11 (*H. haemolyticus*), OTU18 (*Veillonella* sp. oral clone ASCB03), and OTU32 (*Haemophilus parainfluenzae*)). Opposingly, higher intakes of DSLNT was associated with the absence of OTU18 (*Veillonella* sp. oral clone ASCB03) than when this OTU was present.

At day 60, higher intakes of eight individual HMOs (3FL, difucosyllactose (DFLac), DFLNT, DSLNT, LNnT, LNFP I, LNFP II, LNFP III, LSTb and LSTc) were positively associated with different relative abundances of five bacterial OTUs (OTU01 (*S. mitis*), OTU03 (*B. longum* subsp. *infantis*), OTU14 (*V. nakazawae*), OTU18 (*Veillonella* sp. oral clone ASCB03), and OTU32 (*H. parainfluenzae*)). Higher intakes of 3FL and LNFP II were associated with the absence of OTU02 (*S. epidermidis*).

At day 90, higher intakes of five individual HMOs (3′SL (3′-sialyllactose), DFLac, LNFP I, LSTc and DSLNT) were positively associated with different relative abundances of three bacterial OTUs (OTU01 (*S. mitis*), OTU18 (*Veillonella* sp. oral clone ASCB03), and OTU32 (*H. parainfluenzae*)). Higher intakes of six HMOs (6′SL, LNT, LNFP II, LSTb, DSLNH and DSLNT) were associated with the absence of four bacterial OTUs (OTU05 (*S. salivarius*), OTU07 (*G. haemolysans*), OTU19 (*R. mucilaginosa*), and OTU22 (*Bergeyella* sp.).

At day 120, higher intakes of seven individual HMOs (2′FL, 3′SL, 6′SL, LNFP I, LNH, fucodisialyllacto-N-hexaose (FDSLNH), and DSLNH) were positively associated with different relative abundances of four bacterial OTUs (OTU03 (*B. longum* subsp. *infantis*), OTU05 (*S. salivarius*), OTU18 (*Veillonella* sp. oral clone ASCB03), and OTU32 (*H. parainfluenzae*). Higher intakes of four HMOs (2′FL, 6′SL, LNFP I and LNH) were associated with absence of three bacterial OTUs (OTU01 (*S. mitis*), OTU03 (*B. longum* subsp. *infantis*), and OTU18 (*Veillonella* sp. oral clone ASCB03)).

### 2.10. Associations between HMO Intake and Infant Faecal Microbiota

Similar to infant oral samples, individual HMO intakes showed both positive and negative associations with different categories of relative abundance and different bacterial taxa at different time points in infant faecal samples (Table A5).

At day 30, overall, higher intakes of 12 HMOs (3FL, 3′SL, LNnT, LNFP I, LNFP II, LNFP III, LSTb, LSTc, DSLNT, FLNH, difucosyllacto-N-hexaose (DFLNH) and DSLNH) were positively associated with different relative abundances of eight bacterial OTUs (OTU03 (*B. longum subsp. infantis*), OTU05 (*S. salivarius*), OTU10 (*B. pseudocatenulatum*), OTU13 (*Raoultella ornithinolytica*), OTU17 (*Klebsiella pneumoniae*), OTU20 (*B. fragilis*), OTU24 (*B. longum*), and OTU25 (*Parabacteroides distasonis*)). Opposingly, higher intakes of 2′FL, DFLac and DFLNT were associated with the absence of OTU04 (*B. breve*) and a higher intake of 3′SL was associated with the absence of OTU03 (*B. longum* subsp. *infantis*).

At day 60, higher intakes of LNT, LNnT, LNFP I, LNFP II, LNFP III, LSTb, DFLNT, FLNH, LNH, DFLNH were positively associated with different relative abundance of six bacterial OTUs (OTU04 (*B. breve*), OTU06 (*E. coli*), OTU10 (*B. pseudocatenulatum*), OTU17 (*K. pneumoniae*), OTU25 (*P. distasonis*), and OTU29 (*Enterococcus faecalis*)). A higher intake of 3FL was associated with the absence of OTU05 (*S. salivarius*) and a higher intake of FLNH was associated with absence of OTU20 (*B. fragilis*).

At day 90, higher intakes of LNnT, LNFP III, LSTc and FDSLNH were positively associated with different relative abundances of four bacterial OTUs (OTU03 (*B. longum* subsp. *infantis*), OTU13 (*R. ornithinolytica*), OTU25 (*P. distasonis*), and OTU29 (*E. faecalis*)). Higher intakes of 6′SL and DFLNT were associated with absence of OTU24 (*B. longum*) and a higher intake of FLNH was associated with absence of OTU04 (*B. breve*).

At day 120, higher intakes of 2′FL, 3′SL, LNT, LNFP I, LNFP II, DFLNT LNH, FLNH and FDSLNH were positively associated with different relative abundances of six bacterial OTUs (OTU05 (*S. salivarius*), OTU06 (*E. coli*), OTU20 (*B. fragilis*), OTU24 (*B. longum*), OTU25 (*P. distasonis*), and OTU29 (*E. faecalis*)). In contrast, higher intake of 3FL was associated with absence of OTU05 (*S. salivarius*), DFLac with OTU24 (*B. longum*) and OTU25 (*P. distasonis*), DFLNH with OTU14 (*V. nakazawae*), and LNFP II with OTU05 (*S. salivarius*).

## 3. Discussion

This study is one of the few to characterize the temporal development of four inter-related human microbial niches from mother–infant dyads during the exclusive breastfeeding period. As expected, the bacterial profile of each microbiome was unique and changed over time with HMO intakes associating with changes in the infant oral and faecal microbiomes.

During pregnancy the maternal gut microbiota is remodeled with a decrease in bacterial richness during the third trimester and one-month postpartum [38]. Aberrant postpartum microbiota may have negative implications for both mother and infant health [58,59]. Despite this, temporal development of the maternal gut microbiome postpartum has not been well characterized. We observed the most abundant bacterial taxa changing across different time points, with *Dialister invisus* and Bacilli_c;RF39_o;RF39_fa;RF39_ge dominating at day 2–5, *P*. *vulgatus* at months one, two and four, and *P. vulgatus* and *E*. *coli* dominating three-month bacterial profiles. Interestingly, our results differ to those from studies that used strain-level metagenomic profiling at different time points (shortly after delivery within 24 h [13] and at three months [34]), with the exception of *Faecalibacterium prausnitzii*. The difference could be due to geographical and/or dietary variances, as these metagenomic analyses [13,34] were based on Italian mothers and diet [60], lifestyle differences [61], and methodological differences between 16S ribosomal RNA (rRNA) and shotgun sequencing have been shown to be related to the gut microbiome [62].

Significant changes in the prevalence of certain bacterial taxa as well as increases in bacterial richness and Shannon diversity and changes in beta diversity within the maternal microbiota were observed between day 2–5 and one month postpartum. These findings are in stark contrast to previous studies, which have not detected changes, instead reporting stability of diversity and relative distribution of bacterial communities over time [24,39]. The differences observed in this study between first-week and one-month maternal faecal microbiota may be due to a number of factors, including change in maternal diet [63,64,65] as well as the hormonal changes associated with both labour and lactation, such as the dramatic decrease in progesterone and increase in oxytocin and estrogen [66,67]. Additionally, pregnancy and labour are associated with stress, which has been associated with delayed onset of lactation [68] and an increase of intestinal permeability [69], potentially allowing bacteria to travel across the intestinal mucosa. However, we did not analyse pre-pregnancy samples, so it is difficult to know whether the day 2–5 samples are different from the pregnancy sample or not. Future work is needed to prospectively follow women from preconception through pregnancy and into the postpartum period with the addition of documenting maternal measures of metabolic, hormonal, and immune changes together, to assess the impact of pregnancy, birth, and lactation on the maternal gut microbiome.

While previous efforts have been made to characterize the HM bacterial profile using metagenomic [35,37,70,71] and 16S rRNA gene sequencing [14,18,41,42,43,47] methods, only one study has aimed to characterise HM temporal development during the first six months of breastfeeding. William et al. analysed HM bacterial profiles using partial 16S rRNA gene sequencing, at nine different time points, from day two until six months postpartum (52). They reported *Streptococcus* sp. and *Staphylococcus* sp. were dominant and relatively constant over time with less-abundant genera such as *Veillonella* sp., *Propionibacterium* sp., *Prevotella* sp. and *Granulicatella* sp. increasing over time. Our study builds upon this by using full-length 16S rRNA gene sequencing for improved taxonomic resolution. We found 10 bacterial OTUs mapping to *Staphylococcus epidermidis*, *Streptococcus salivarius*, *Streptococcus mitis*, *Gemella haemolysans*, *Streptococcus agalactiae*, *Cutibacterium acnes*, *Acinetobacter johnsonii*, *Moraxella osloensis*, *Streptococcus lactarius* and *Streptococcus anginosus* dominated HM bacterial profiles and that the most abundant OTUs changed over time.

Previous studies documenting differences in HM bacteria with lactation stage have mainly focused on the transition from colostrum to mature milk [14,19,44,45]. By extending the time of sample collection, we found that some abundant species, such as *S. epidermidis,* were more prevalent in the first month postpartum compared to two and four months. *S. epidermidis* is a ubiquitous commensal of the skin and mucosal environments [72,73] and has emerged as the predominant pathogen of sepsis in preterm infants [74]. The conversion of *S. epidermidis* from commensal skin inhabitant to a virulent pathogen may be due to disruption of the skin epithelial barrier or through selective pressure due to extensive use of antibiotics in preterm infants [75]. However, in healthy term infants, *S. epidermidis* primarily plays a commensal role by inhibiting virulent pathogens and educating and stimulating the innate immune system [75]. Indeed, a recent study has shown that the gut and skin of term neonates were colonized with strains of *S. epidermidis* genetically similar to those present in HM [76], supporting a role for HM bacteria in infant microbiome colonization. Our data suggest that bacteria in HM are temporally dynamic, meaning that infants are exposed to a differing combination of bacteria across time. While the composition of the HM microbiome differed over time, we did not observe differences in richness and Shannon diversity over time, similar to a previous study [77] but in contrast to another [44]. This suggests that geographic, genetic, and dietary factors may influence bacterial community structure in milk.

The oral cavity serves as an initial entry point for colonization of the oral and gut microbiota [78,79], and thus, the oral microbiota influences infant health [79] with early life dysbiosis being related to conditions such as dental caries, periodontitis and oral mucosal diseases [80,81]. As such, characterisation of oral temporal development during the exclusive breastfeeding period is important to identify early factors that may disturb optimal colonisation. We found that 11 bacterial OTUs (*S. epidermidis*, *S. salivarius*, *S. mitis*, *G. haemolysans*, *V. nakazawae*, *B. longum* subsp. *infantis*, *H. haemolyticus*, *R. mucilaginosa*, *Bergeyella* sp., *H. parainfluenzae* and *Veillonella* sp. oral clone ASCB03) dominated the infant oral microbiota. Our findings are in agreement with a US study that found a core microbial community of the *S. mitis* group, *R. mucilaginosa*, *S. salivarius*, and *G. haemolysans* in the first three months of life [27]. However, we did not observe the previously reported *Veillonella parvula* group and *Veillonella* HB016 [27]. The differences between studies could be due to host genetic variations, ethnicity and geographical location, as these have been shown to influence the oral microbiota [82,83,84].

We also found that the relative abundance and prevalence of these OTUs in the infant oral cavity changed over time. *S. mitis* was one of the most abundant and ubiquitous OTUs, whose relative abundance more than doubled from day 2–5 to all other time points. *S. mitis* is major oral organism and is likely to modulate oral colonization of other bacterial species, as demonstrated in previous studies [85,86]. Additionally, the infant oral cavity contains high levels of the metabolites xanthine and hypoxanthine [87]. An in vitro study that added these metabolites to HM showed production of hydrogen peroxide. If this translates to an in vivo setting, this may in turn inhibit the growth of opportunistic pathogens such as *Staphylococcus aureus*, *Pseudomonas aeruginosa* and *Salmonella* spp. [86,88]. This could help explain our observation of *S. epidermidis* being dominant in the infant oral microbiota after birth, but reducing to <1% over the first month and then being absent at four months. A recent in vitro study by Sweeney et al. reported immediate inhibition of *S. epidermidis* when a saliva-HM mixture was supplemented with hypoxanthine and xanthine [89]. Future studies investigating the temporal development of oral microbiota should also consider analyzing oral bacterial metabolites as they may influence oral colonization.

Conflicting data exist with respect to the diversity of the infant oral microbiome. For example, we found that neither richness nor Shannon diversity in infant oral samples differed over the first four months of life. Similarly, Hurley et al. reported that Shannon diversity remained stable with increasing age [90], while in contrast, Sulyanto et al. reported Shannon diversity increased over time [27]. This result might be confounded by the feeding methods used, as three infants were solely breastfed, one solely formula-fed, and five were mix-fed [27]. Previous longitudinal studies have shown bacterial richness increases and the compositional profile changes with age between three months and seven years [31,91,92,93]; however, this is also likely to be due to consumption of food and fluids other than HM [27,94]. Our beta diversity analysis showed diversity significantly increased from day 2–5 to one, two, three and four months, highlighting the continuous development and maturation of the oral microbiota in infancy.

Infant oral microbiota are important for oral health [79] and differences in the oral microbiota between breastfed and formula-fed infants have been reported [91,95,96], however, little is known about how HMOs impact the oral microbiota. A recent in vitro study assessed the effect of 2′FL and galacto-oligosaccharides on the growth and adhesion characteristics of the caries-associated oral pathogen *Streptococcus mutans* and reported 2′FL both limits growth and inhibits adhesion of *S. mutans* to saliva-coated hydroxyapatite [97]. However, there is an absence of information on how daily intakes of HMOs influence infant oral microbiota. The current study is the first to provide an insight into associations between daily intakes of various HMOs and infant oral bacteria; however, these associations differed over time (Table A4). Additionally, we observed several individual HMO intakes supported the growth of infant oral bacteria such as *Veillonella* spp., *S. mitis* and *S*. *salivarius,* which are common oral cavity inhabitants [98,99,100]. Furthermore, we observed intakes of 3FL and LNFP II were negatively associated with abundance of *S. epidermidis* in the infant oral cavity. *S. epidermidis* is one of the most abundant colonizers of skin and mucosal surfaces [101] and is associated with dental caries [102]. Therefore, the results from the current study may suggest a protective role of HMOs in the infant oral cavity as well as involvement of HMOs in establishment of the infant oral microbiota. However, results from the current study need to be confirmed in larger longitudinal cohorts.

The early life infant gut microbiota is often regarded as having high plasticity due to its low diversity and rapid development [103]; however, neither have been extensively studied during the exclusive breastfeeding period. Carrothers et al. found *Bacteroides* sp., *Faecalibacterium* sp., *Lachnospiraceae incertae sedis* sp. and *Prevotella* sp. dominated the infant faecal microbiota from day two to six months, while *Bifidobacterium* sp. only made up a small proportion [39]. Contrarily, we observed that *Bifidobacterium* species including *B. longum* subsp. *infantis*, *B. breve*, *B. pseudocatenulatum* and *B. longum* dominated the bacterial profile over the first four months of life. Previous studies using strain-level analyses during the first year of life have reported that the breastfed infant gut is dominated by *Bifidobacterium* species, including *B. longum* subsp. *longum*, *B. breve*, *B. bifidum*, *B. longum* subsp. *infantis*, *B. adolescentis* and *B. pseudocatenulatum* [9,34,35,37,104]. In comparison, formula-fed infants have been found to have a more diverse bacterial community and higher abundance of *Clostridium difficile*, *Granulicatella adiacens*, *Citrobacter* spp., *Enterobacter cloacae* and *Bilophila wadsworthia* [105]. The high levels of *Bifidobacterium* spp. in breastfed infants are likely due to HMOs, which promote the growth of this genera [106,107,108,109]. *Bifidobacterium* spp. are early colonisers of the infant gut, and producers of aromatic lactic acids, such as indole lactic acid, which modulate intestinal immune responses via their interaction with the aryl hydrocarbon receptor [110,111]. This emphasizes the role of Bifidobacterial priming and programming of immune functionality in early life. We also observed no change in richness, Shannon diversity and beta diversity over time in infant faecal samples. However, others have observed changes [112,113], that may be associated with the introduction of solids and/or cessation of breastfeeding, promoting the survival and proliferation of varied types of microbial species [105,114]. A greater understanding of the temporal development of the gut during early life may identify signatures that are less favourable to positive health outcomes and thus enable development of potential interventions to improve short- and long-term health.

One of the potent factors shaping the temporal development of exclusively breastfed infant gut bacterial profiles are HMOs, the third most abundant component of HM, that acts as a prebiotic for bacterial colonisation in the infant gut amongst other functions [115,116,117]. It has been reported that most HMOs are non-digestible and reach the colon undigested [118,119], where they serve as a carbon source for bacterial fermentation [120,121] and are involved in host–microbe interactions [122]. Substantial evidence exists showing associations between HMO concentrations and infant gut bacteria including *Bifidobacterium* sp., *Lactobacillus* sp., *Bacteroides* sp., *Veillonella* sp., *Enterococcus* sp. and *Streptococcus* sp. [53,55,123,124]. However, no study to date has evaluated the impact of HMO daily intakes on infant gut microbiota. A novel finding of this study is a detection of both positive and negative associations between various individual HMO daily intakes and infant gut bacteria (*B. longum* subsp. *infantis*, *S. salivarius*, *E. coli*, *B. pseudocatenulatum*, *R. ornithinolytica*, *K. pneumoniae*, *B. fragilis*, *B. longum*, *P. distasonis*, and *E. faecalis)*. However, these associations differed over time. For example, at one-month intakes of 2′FL, DFLac and DFLNT were negatively associated *B. breve*, while intake of DFLNH was positively associated at two months, and intake of FLNH negatively associated at three months. The differences between different time points could be a result of concentrations of HMOs significantly changing over time [125], thereby influencing HMO daily intakes. In addition to HMOs, there are likely other mechanisms influencing microbial community structure. For example, the presence of other components in HM such as total protein [53], lysozyme [126], secretory immunoglobulins [127] and other endogenous factors have been shown to influence the infant gut microbiota.

One possible explanation for associations of infant gut *Bifidobacterium* spp. and *Bacteroides* spp. with certain HMO intakes in our study could be due to their genetic material. Marcobal et al. analyzed the genomes of 16 bacterial strains of gut microbiota and reported *Bacteroides fragilis*, *Bacteroides vulgatus*, *Bifidobacterium infantis* and *Bifidobacterium longum* have different genes coding for production of enzymes such as α-galactosidase, β-N-acetylgalactosaminidase, β-hexosaminidase, α-L-fucosidase, sialidase, β-galactosidase, and α1,2-L-fucosidase for glycoside hydrolases of HMOs [52]. However, the reason for HMOs associations with other gut bacteria is unclear. It is plausible that other gut bacteria have been directly utilizing the HMOs or they were benefitting from cross-feeding of HMOs fermented by *Bifidobacterium* spp. and *Bacteroides* spp [108,128]. Moreover, these bacteria are able to convert HMOs into short-chain fatty acids such as lactate, acetate, propionate and butyrate [129], which serve as nutrients for cross-feeding between gut bacteria [130]. The advantage of *Bifidobacterium* spp. and *Bacteroides* spp. of HMO utilization may promote diversity and dominance of these bacteria during early life while down regulating the colonization of other taxa. Additionally, HMOs act as receptor decoys and prevent the binding of Clostridia, *Campylobacter*, and the stable toxin of entero-toxigenic *E. coli* to their target host cell receptors [131], thus limiting their colonization in the infant gut. However, future studies are required to analyze the metagenome of gut microbiota to identify if other gut bacteria contain genes that allow the production of enzymes which could help to utilize HMOs.

HMO composition varies between mothers and is dependent on maternal genetics [132,133], with many HMOs significantly decreasing in concentration over the first two years of lactation [125,134]. Indeed, we found that the concentrations of 13 HMOs in secretor mothers and four HMOs in non-secretor mothers decreased across the first four months, except for 3FL, which increased in concentration in both secretor and non-secretor mothers as lactation progressed, which is consistent with reports by Plows et al. [125]. Additionally, a novel aspect of this study was documenting changes in the daily intakes of HMOs over the first four months of life in infants born to secretor and non-secretor mothers. However, calculation of daily intakes gave differing results, with 11 individual HMO intakes of infants born to secretor mothers and three HMO intakes of infants from non-secretor mothers decreasing from month one to four. The daily intake of 3FL was however significantly higher across the same period for infants of both secretor and non-secretor mothers. To date only one study has measured HMO intakes and compared intake differences between infants born to mothers with normal weight, over-weight and obesity status [135]. They reported infants born to mothers with obesity had lower intakes of LNH, FLNH, DFLNH, DFLNT, and DSLNH compared to infants born to mothers with normal weight and over-weight status. Although these results are not comparable to our study, they provide evidence of maternal influence on infant HMO intakes.

The strengths of this study include the exclusivity of breastfeeding, full-length 16S rRNA gene sequencing and measurement of daily HMO intakes. The limitations include low participant numbers (*n* = 10) which did not allow for stratification based on maternal secretor status for the analysis of associations between HMO intakes and the infant microbiomes, therefore results should be interpreted cautiously. Despite achieving high sequencing read numbers, sequencing coverage for maternal faecal samples was greatly reduced compared to coverage values for human milk, infant oral and infant faecal samples. This indicates that microbial diversity in these samples is higher than reported here and is likely due to sequencing low (human milk, infant oral and infant faecal) and high (maternal faecal) biomass samples together. Future studies should consider separately preparing low and high biomass sample libraries for sequencing in order to ensure appropriate sequencing coverage across all samples. We do note though, that although this is a limitation of the current study, the samples relevant to the primary aim, describing longitudinal development of the infant gut microbiome, all have sufficient sequencing coverage. Further, our population consisted of vaginally delivered term, healthy, exclusively breastfed infants from Caucasian mothers of high social-economic status living in Australia; therefore, the results may not be transferable to other populations. Nonetheless, this study has yielded important findings that warrant validation in larger longitudinal cohorts with extensive sampling.

## 4. Materials and Methods

### 4.1. Study Design

Participants were recruited during the third trimester of pregnancy (>30 weeks gestation) to participate in the BLOSOM (Breastfeeding Longitudinal Observational Study of Mothers and kids) study, as previously described [136]. In this sub-study, 10 mother–infant pairs were chosen based on the following additional criteria; healthy women with no major pregnancy complications, vaginal birth, term infant, exclusively breastfeeding, no maternal smoking, no maternal or infant antibiotic use during labour or in the first four months postpartum, no maternal nipple pain, no infant pacifier use in the first 5 days of life, and no solid introduction before four months of age. The study was approved by the Human Research Ethics Committee at The University of Western Australia (RA/4/20/4023); all participants provided informed written consent to participate.

### 4.2. Sample and Data Collection

Mothers answered a background questionnaire at the time of recruitment and collected their own and their infant’s samples during the study at five time points: 2–5, 30, 60, 90 and 120 days postpartum.

Mothers selected one breast from which to donate HM samples throughout the study and were asked not to breastfeed or express milk from the breast for at least two hours prior to sample collection. Mothers washed their hands thoroughly with soap and water and wore gloves during sample collection. The nipple and areola of the expressing breast were cleaned with prep pads (70% isopropyl alcohol and 2% chlorhexidine digluconate, Reynard Health Supplies, Artarmon, NSW, Australia), followed by rinsing with sterile saline solution (Livingstone, Mascot, NSW, Australia) and drying with sterile gauze swabs (Livingstone, Mascot, NSW, Australia). Up to 20 mL (otherwise as much as possible) of HM was expressed directly into sterile tubes using hand-expression, as previously described [137].

Maternal faecal samples were collected from toilet paper using an E-swab (Becton, Dickinson and Company, Franklin Lakes, NJ, USA). Infant faecal samples were collected from diapers within 1–2 h post-bowel movement using an E-swab and avoiding any urine. For infant oral samples, the E-swab was firmly rubbed up and down and in a circular motion against the inside of the cheek 10 times. Using the same E-swab, the process was repeated on the other side. E-swabs were carefully removed from the mouth without touching the lips or other surfaces and were preserved in 1 mL liquid Amies medium.

All samples were stored in the refrigerator at the participant’s home for up to 18 h before being collected and transported on ice to the laboratory, where they were immediately aliquoted into sterile tubes (Sarstedt, Numbrecht, Germany) and stored at −80 °C until further analysis. All samples collected by E-swab were eluted into the collection media by vortexing for 5 s prior to aliquoting.

### 4.3. Human Milk Oligosaccharides Analysis

100 μL of HM aliquots from each participant (*n* = 10) per time point were sent on dry ice to the Bode Lab at the University of California, (San Diego, CA, USA). The concentration and composition of HMOs in HM samples was analyzed by HPLC after labelling with the fluorescent tag 2-aminobenzamide as described previously [138]. The following 19 HMOs were identified and quantified: 2′FL, 3FL, 3′SL, 6′FL, DFLac, DFLNH, DFLNT, DSLNH, DSLNT, FDSLNH, FLNH, LNFP I, LNFP II, LNFP III, LNH, LNnT, LNT, LSTb and LSTc. Maternal secretor status was identified based on the presence or near-absence of 2′FL in HM.

### 4.4. 24-h Milk Intake

Infant 24-h milk intake was measured at the three month time point by mothers in their homes using the 24-h milk profile protocol as described previously [139]. Briefly, mothers weighed their infant before and after each feed on electronic scales (±2.0 g; Electronic Baby Weigh Scale, Medela Inc., McHenry, IL, USA). HM intake (g) was calculated by subtracting the weight of the infant before the feed from the weight after the feed. Three months 24-h milk intakes were considered representative of intakes during the exclusive breastfeeding period as there is no significant variation in HM intake from one to six months within infants [140].

### 4.5. Daily Intakes of HMOs

HMO daily intakes (µg) were determined as the concentration of HMOs (µg/mL) multiplied by 24-h milk intake (grams).

### 4.6. DNA Extraction and Quantification

One mL aliquots of HM were centrifuged at 40,000× *g* for 5 min at 4 °C. The supernatant and lipid fraction were discarded. Maternal faecal, infant faecal, and infant oral samples were centrifuged at 40,000× *g* for 5 min at 4 °C and the supernatant was discarded. For all sample types, DNA was extracted from the cell pellet using the QIAGEN MagAttract Microbial DNA Isolation Kit (QIAGEN, Hilden, Germany) on the Kingfisher Flex platform, following the manufacturer’s instructions. Two negative extraction controls each consisting of 1 mL sterile nuclease-free water (Integrated DNA Technologies, Coralville, IA, USA) were included at the centre of each 96-well extraction plate.

Total DNA yield was assessed using the Qubit^®^ dsDNA High Sensitivity Assay (Invitrogen, Mulgrave, VIC, Australia) on a Qubit^®^ 2.0 Fluorometer (Life Technologies, Mulgrave, VIC, Australia) according to the manufacturer’s instructions. The limit of detection was 10 pg/μL.

### 4.7. 16S rRNA Gene Amplification and Barcoding

The full-length 16S rRNA gene was amplified using the primer pair 27F and 1492R with a universal UNITAG sequence and amine block attached to the 5′ ends of each primer, as previously described [141,142].

Primary PCR was carried out in 25 µL reactions containing 0.3 μM each of the forward and reverse primers, 1X AccuStart II ToughMix (Quantabio, Beverly, MA, USA), 0.625 µL each of ArcticZymes dsDNase and DTT (ArcticZymes PCR decontamination kit, Tromsø, Norway), 5.5 µL nuclease-free water and 5 µL of template or nuclease-free water. The activation and inactivation of ArcticZymes dsDNase was performed as described previously [142]. Two negative template controls were included for every 94 samples. The PCR cycling conditions consisted of an initial heating step at 94 °C for 3 min, followed by 35 cycles of 94 °C for 30 s, 52 °C for 30 s, and 72 °C for 2 min and a final extension step of 72 °C for 5 min. Primary PCR products were visualized on a QIAxcel capillary gel electrophoresis system using a DNA high-resolution cartridge (run parameters OM500) to confirm the presence and size of amplicons. Primary PCR products were purified using NucleoMag NGS magnetic beads (Macherey-Nagel, Düren, Germany), normalized to 1 ng/µL and used as template for the barcoding PCR.

Primary PCR products were barcoded using an asymmetric barcoding strategy. PacBio UNITAG barcoded primers 1F–8F and 16R–30R were used. PCR reactions were carried out in 20 µL volumes containing 0.3 μM each of the forward and reverse barcoded primers, 1X AccuStart II ToughMix, and 2 µL of template or nuclease-free water (negative template control). PCR cycling conditions were the same as described above, but with 20 cycles.

Barcoded PCR amplicons were pooled in equimolar concentrations based on QIAxcel quantification of the target ~1500 bp band. The pools were gel purified using a QIAquick gel extraction kit (QIAGEN) according to manufacturer’s protocol. ~500 ng of DNA (pooled amplicons) was used for library preparation and sequencing.

### 4.8. PacBio Sequencing

Purified amplicon pools were sequenced at the Australian Genome Research Facility (AGRF) at The University of Queensland, QLD, Australia. SMRTbell adapters were ligated onto the barcoded PCR products and the libraries were sequenced by Pacific Biosciences single molecule real-time (SMRT) high-fidelity (HiFi) sequencing on two SMRT cells using the PacBio Sequel II System. Raw data were processed using PacBio SMRTLink to generate demultiplexed .fastq files.

### 4.9. Sequencing Data Processing

Full-length 16S rRNA gene sequence data were processed using Mothur v.1.44.3 [143] (as previously described [137]) on the Pople supercomputer at a high-performance computing cluster (Karton, A; The University of Western Australia). Briefly, .fastq files were converted to .fasta files and merged into a single .fasta file. The merged .fasta file was length filtered (1336–1743 bp) and sequences containing homopolymers of >9 bases were removed. Sequences were aligned to the SILVA reference alignment v138 and pre-clustered. Chimeric sequences were removed using the chimera.vsearch command. Sequences were then classified using classify.seqs with the SILVA taxonomy database v138 and a confidence threshold of 80. Based on classification, non-bacterial sequences were filtered and discarded from the dataset. Operational taxonomic units (OTUs) were created using the cluster.split command with a 0.03 similarity cut-off value. Clustered OTUs were assigned taxonomy using classify.otu. The Good’s coverage for each sample type for raw sequencing data and sequencing depth at 427 reads is provided in Table A1. Subsampling was performed at 427 reads based on an average Good’s coverage (collectively for all samples) value of 72.0%, eliminating four samples: Mother (M) 1 day 30 maternal faecal sample (20 reads), M1 day 60 maternal faecal sample (297 reads), M2 day 2–5 maternal faecal sample (16 reads), and M6 day 2–5 HM sample (62 reads). The subsampled data at 427 reads were used for all downstream analyses. Reads from negative extraction controls and negative PCR controls are provided in Table A2.

### 4.10. Statistical Analysis

Data were analysed and graphs generated using the R environment for statistical computing [144,145,146]. Maternal and infant demographics are provided as mean ± standard deviation (minimum–maximum) or *n* (%).

Alpha diversity was assessed using the Shannon Index and richness (number of different OTUs). In order to compare across time points and samples, linear mixed models were performed with outcomes of Shannon Index and richness, fixed factors of sample (HM, maternal faecal, infant oral and infant faecal), time (days 2–5, 30, 60, 90 and 120), and their respective interaction, as well as a random effect of participant. Estimated mean differences (MDs), 95% confidence intervals (CIs) and *p-*values are provided.

Beta diversity was assessed by performing a PERMANOVA on the Bray-Curtis dissimilarity matrix. Fixed factors of sample, time, and their respective interaction were included in the model, as well as a random effect of participant. *p*-values are provided. In order to visualise the dissimilarities, a non-metric multi-dimensional scaling (NMDS) plot is presented which was developed using the Bray–Curtis dissimilarity matrix.

Relative abundances were categorised as Absent (relative abundance of 0), Low (>0 to <0.05), Medium (0.05 to 0.3) and High (>0.3). Wilcoxon signed-rank tests for paired data, with the Pratt correction for ties, were used to compare this categorised relative abundance variable between time points. The modelling considered only samples that had paired data for the pairwise comparison of interest, and at least one sample’s measures must have differed between the two time points being compared. Additionally, only OTUs that were present at an average relative abundance of >1% were considered. *p*-values are provided, as well as a heat-map to visualise the changes in abundance over time.

Linear mixed models were used to assess whether concentrations and intakes of each HMO differed over time and between maternal secretor status. Fixed effects of time and secretor status were included, along with their interactions, as well as a random effect of participant. Contrasts were examined for all pairwise comparisons with Tukey corrections.

To assess the relationship between relative abundances in both infant faecal and infant oral samples with HMO intakes, an ANOVA was performed at each time point with the categorised relative abundance data. Estimated mean differences (MD), standard errors (SE) and *p*-values are provided. Significance for all analyses was considered at the 5% level.

## 5. Conclusions

In conclusion, we found the maternal gut, HM and infant oral and gut microbiota are dominated by a small number of bacterial taxa that changed in relative abundance over the first four months of life. Furthermore, the variations in infant oral and faecal bacterial profiles were associated with HMO intakes, the clinical relevance of which, however, is currently unknown, but may have implications for infant microbiota development.

## Figures and Tables

**Table 1:** Maternal and infant characteristics (*n* = 10).

Characteristics (*n* = 10)	Mean ± SD (Min–Max) or *n* (%)
Maternal	
Age at infant birth (years)	31.60 ± 2.42 (28–35)
Parity	2.50 ± 0.50 (2–3)
Infant	
Male (*n*, (%))	4 (40.0%)
Gestational age (weeks)	39.26 ± 1.11 (36.6–40.2)
Birth weight (grams)	3622.50 ± 234.11 (3320–4020)
Birth length (cm)	51.55 ± 1.39 (49–54)
24-h milk intake (grams)	837.80 ± 131.17 (580–1040)

SD—standard deviation; Min—minimum; Max—maximum; %—percentage; n—number.

**Table 2:** Mean differences (MD), 95% confidence intervals (CIs) and *p*-values for pairwise comparisons of alpha diversity measures (Richness and Shannon diversity) between time points, for each sample type.

Time Points	Maternal Faecal		Human Milk		Infant Oral		Infant Faecal	
	MD (95% CI)	*p-*Value	MD (95% CI)	*p*-Value	MD (95% CI)	*p*-Value	MD (95% CI)	*p*-Value
	Richness (Observed OTUs)							
2–5 days vs. 30 days	−72.27 (−118.35, −26.19)	**0.0023**	−4.18 (−49.06, 40.7)	0.8543	33.3 (−10.35, 76.95)	0.1339	−3.9 (−47.55, 39.75)	0.8602
2–5 days vs. 60 days	−44.82 (−90.9, 1.26)	0.0565	−3.58 (−48.46, 41.3)	0.8750	36.3 (−7.35, 79.95)	0.1025	−14.7 (−58.35, 28.95)	0.5070
2–5 days vs. 90 days	−71.68 (−116.55, −26.8)	**0.0019**	−7.28 (−52.16, 37.6)	0.7491	32.4 (−11.25, 76.05)	0.1447	3.2 (−40.45, 46.85)	0.8851
2–5 days vs. 120 days	−60.98 (−105.85, −16.1)	**0.0080**	−32.28 (−77.16, 12.6)	0.1574	33.6 (−10.05, 77.25)	0.1305	−17.5 (−61.15, 26.15)	0.4297
30 days vs. 60 days	27.44 (−18.56, 73.45)	0.2406	0.6 (−43.05, 44.25)	0.9784	3 (−40.65, 46.65)	0.8922	−10.8 (−54.45, 32.85)	0.6258
30 days vs. 90 days	0.59 (−44.29, 45.47)	0.9793	−3.1 (−46.75, 40.55)	0.8887	−0.9 (−44.55, 42.75)	0.9676	7.1 (−36.55, 50.75)	0.7485
30 days vs. 120 days	11.29 (−33.59, 56.17)	0.6200	−28.1 (−71.75, 15.55)	0.2055	0.3 (−43.35, 43.95)	0.9892	−13.6 (−57.25, 30.05)	0.5393
60 days vs. 90 days	−26.85 (−71.73, 18.02)	0.2392	−3.7 (−47.35, 39.95)	0.8673	−3.9 (−47.55, 39.75)	0.8602	17.9 (−25.75, 61.55)	0.4193
60 days vs. 120 days	−16.15 (−61.03, 28.72)	0.4783	−28.7 (−72.35, 14.95)	0.1960	−2.7 (−46.35, 40.95)	0.9029	−2.8 (−46.45, 40.85)	0.8994
90 days vs. 120 days	10.7 (−32.95, 54.35)	0.6290	−25 (−68.65, 18.65)	0.2598	1.2 (−42.45, 44.85)	0.9568	−20.7 (−64.35, 22.95)	0.3505
	**Shannon diversity**							
2–5 days vs. 30 days	−0.82 (−1.61, −0.02)	**0.0435**	−0.11 (−0.88, 0.66)	0.7775	0.5 (−0.25, 1.25)	0.1909	0.06 (−0.69, 0.81)	0.8763
2–5 days vs. 60 days	−0.54 (−1.33, 0.25)	0.1824	−0.19 (−0.96, 0.58)	0.6237	0.6 (−0.15, 1.35)	0.1168	−0.32 (−1.08, 0.43)	0.3936
2–5 days vs. 90 days	−0.92 (−1.69, −0.15)	**0.0199**	−0.27 (−1.04, 0.5)	0.4857	0.61 (−0.14, 1.36)	0.1093	0.15 (−0.6, 0.9)	0.7030
2–5 days vs. 120 days	−0.71 (−1.49, 0.06)	0.0691	−0.69 (−1.46, 0.09)	0.0809	0.55 (−0.2, 1.3)	0.1516	−0.2 (−0.95, 0.55)	0.5983
30 days vs. 60 days	0.28 (−0.51, 1.07)	0.4876	−0.08 (−0.83, 0.67)	0.8305	0.1 (−0.65, 0.85)	0.7927	−0.38 (−1.13, 0.37)	0.3134
30 days vs. 90 days	−0.1 (−0.87, 0.67)	0.7937	−0.16 (−0.91, 0.59)	0.6697	0.11 (−0.64, 0.86)	0.7671	0.09 (−0.66, 0.84)	0.8215
30 days vs. 120 days	0.1 (−0.67, 0.87)	0.7962	−0.58 (−1.33, 0.17)	0.1319	0.05 (−0.7, 0.8)	0.8989	−0.26 (−1.01, 0.49)	0.4950
60 days vs. 90 days	−0.38 (−1.15, 0.39)	0.3309	−0.08 (−0.83, 0.67)	0.8316	0.01 (−0.74, 0.76)	0.9734	0.47 (−0.28, 1.22)	0.2178
60 days vs. 120 days	−0.18 (−0.95, 0.59)	0.6499	−0.49 (−1.24, 0.26)	0.1954	−0.05 (−0.8, 0.7)	0.8920	0.12 (−0.63, 0.87)	0.7438
90 days vs. 120 days	0.2 (−0.55, 0.95)	0.5933	−0.41 (−1.16, 0.34)	0.2786	−0.06 (−0.81, 0.69)	0.8657	−0.35 (−1.1, 0.4)	0.3642

*p*-values significant at the 5% level are highlighted in bold text.

**Table 3:** PERMANOVA results assessing the beta diversity (Bray–Curtis dissimilarity) between time points, for each sample type.

Time Points	Maternal Faecal	Human Milk	Infant Oral	Infant Faecal
	Bray-Curtis Dissimilarity			
2–5 days vs 30 days	**0.0365**	0.4227	**0.0029**	0.3971
2–5 days vs. 60 days	0.1037	0.1354	**0.0211**	0.4323
2–5 days vs. 90 days	0.3725	0.3969	**<0.0001**	0.1590
2–5 days vs. 120 days	0.8534	0.2948	**0.0043**	0.4046
30 days vs. 60 days	0.6497	**0.0289**	0.6791	0.4193
30 days vs. 90 days	0.3358	0.0741	**0.0081**	0.2825
30 days vs. 120 days	0.3102	0.0513	**0.0415**	0.5278
60 days vs. 90 days	0.4660	0.6015	0.1343	0.2471
60 days vs. 120 days	0.5362	0.2185	0.6746	0.5739
90 days vs. 120 days	0.6205	0.5657	0.2726	0.3194

*p*-values significant at the 5% level are highlighted in bold text.

**Table 4:** Mean differences (MD), 95% confidence intervals (CIs) and *p-*values for pairwise comparisons of alpha diversity measures (Richness and Shannon diversity) between sample types, at each time point.

Sample Types	Day 2–5		Day 30		Day 60		Day 90		Day 120	
	MD (95% CI)	*p*-Value	MD (95% CI)	*p*-Value	MD (95% CI)	*p*-Value	MD (95% CI)	*p*-Value	MD (95% CI)	*p*-Value
	Richness (Observed OTUs)									
HM vs. IF	7.92 (−36.96, 52.8)	0.7280	8.2 (−35.45, 51.85)	0.7112	−3.2 (−46.85, 40.45)	0.8851	18.4 (−25.25, 62.05)	0.4064	22.7 (−20.95, 66.35)	0.3060
HM vs. IO	−64.08 (−108.96, −19.2)	**0.0054**	−26.6 (−70.25, 17.05)	0.2306	−24.2 (−67.85, 19.45)	0.2753	−24.4 (−68.05, 19.25)	0.2713	1.8 (−41.85, 45.45)	0.9352
HM vs. MF	−127.81 (−173.89, −81.72)	**<0.0001**	−195.89 (−240.77, −151.01)	**<0.0001**	−169.05 (−213.92, −124.17)	**<0.0001**	−192.2 (−235.85, −148.55)	**<0.0001**	−156.5 (−200.15, −112.85)	**<0.0001**
IF vs. IO	−72 (−115.65, −28.35)	**0.0014**	−34.8 (−78.45, 8.85)	0.1174	−21 (−64.65, 22.65)	0.3436	−42.8 (−86.45, 0.85)	0.0546	−20.9 (−64.55, 22.75)	0.3458
IF vs. MF	−135.72 (−180.6, −90.85)	**<0.0001**	−204.09 (−248.97, −159.21)	**<0.0001**	−165.85 (−210.72, −120.97)	**<0.0001**	−210.6 (−254.25, −166.95)	**<0.0001**	−179.2 (−222.85, −135.55)	**<0.0001**
IO vs. MF	−63.72 (−108.6, −18.85)	**0.0057**	−169.29 (−214.17, −124.41)	**<0.0001**	−144.85 (−189.72, −99.97)	**<0.0001**	−167.8 (−211.45, −124.15)	**<0.0001**	−158.3 (−201.95, −114.65)	**<0.0001**
	**Shannon diversity**									
HM vs. IF	−0.06 (−0.83, 0.71)	0.8746	0.11 (−0.64, 0.86)	0.7765	−0.19 (−0.94, 0.56)	0.6090	0.36 (−0.39, 1.11)	0.3498	0.42 (−0.33, 1.17)	0.2666
HM vs. IO	−1.21 (−1.98, −0.44)	**0.0023**	−0.6 (−1.35, 0.15)	0.1160	−0.42 (−1.17, 0.33)	0.2718	−0.33 (−1.08, 0.42)	0.3931	0.02 (−0.73, 0.77)	0.9512
HM vs. MF	−1.95 (−2.74, −1.16)	**<0.0001**	−2.65 (−3.42, −1.88)	**<0.0001**	−2.29 (−3.06, −1.52)	**<0.0001**	−2.59 (−3.34, −1.84)	**<0.0001**	−1.98 (−2.73, −1.23)	**<0.0001**
IF vs. IO	−1.15 (−1.9, −0.4)	**0.0029**	−0.71 (−1.46, 0.04)	0.0640	−0.22 (−0.97, 0.53)	0.5559	−0.68 (−1.43, 0.07)	0.0747	−0.4 (−1.15, 0.35)	0.2937
IF vs. MF	−1.89 (−2.66, −1.12)	**<0.0001**	−2.76 (−3.53, −1.99)	**<0.0001**	−2.1 (−2.87, −1.33)	**<0.0001**	−2.95 (−3.7, −2.2)	**<0.0001**	−2.4 (−3.15, −1.65)	**<0.0001**
IO vs. MF	−0.74 (−1.51, 0.03)	0.0603	−2.05 (−2.82, −1.28)	**<0.0001**	−1.87 (−2.65, −1.1)	**<0.0001**	−2.27 (−3.02, −1.52)	**<0.0001**	−2 (−2.75, −1.25)	**<0.0001**

HM—human milk; IF—infant faecal; IO—infant oral; MF—maternal faecal. *p*-values significant at the 5% level are highlighted in bold text.

**Table 5:** PERMANOVA results assessing the beta diversity (Bray-Curtis dissimilarity) between sample types at each time point.

Sample Types	Day 2–5	Day 30	Day 60	Day 90	Day 120
	Bray-Curtis Dissimilarity				
Human milk vs. Infant faecal	**<0.0001**	**<0.0001**	**<0.0001**	**<0.0001**	**<0.0001**
Human milk vs. Infant oral	**0.0218**	**0.0001**	0.0563	**0.0004**	**<0.0001**
Human milk vs. Maternal faecal	**0.0004**	**<0.0001**	**0.0002**	**<0.0001**	**<0.0001**
Infant faecal vs. Infant oral	**<0.0001**	**0.0006**	**<0.0001**	**<0.0001**	**<0.0001**
Infant faecal vs. Maternal faecal	**0.0002**	**<0.0001**	**<0.0001**	**<0.0001**	**<0.0001**
Infant oral vs. Maternal faecal	**<0.0001**	**<0.0001**	**0.0069**	**<0.0001**	**<0.0001**

*p*-values significant at the 5% level are highlighted in bold text.

## Data Availability

The data presented in this study are available from the corresponding author upon reasonable request.

## Appendix A

**Table A1:** Good’s coverage for each sample type.

Sample Types	Good’s Coverage (Raw Sequencing Data)	Good’s Coverage (Sequencing Depth 427)
Maternal faecal	43.0% (0.0–91.6%)	38.7% (17.7–89.8%)
Human milk	88.6% (71.3–98.1%)	82.0% (58.2–97.2%)
Infant oral	86.0% (62.6–93.9%)	78.3% (53.5–90.3%)
Infant faecal	91.4% (79.1–96.5%)	85.1% (68.1–93.4%)

The data is presented as median (min–max).

**Table A2:** Number of reads of bacterial OTUs detected in negative extraction controls (*n* = 6) and negative PCR control (*n* = 8).

OTUs	Genus	EC 1	EC 2	EC 3	EC 4	EC 5	EC 6	PCRC 1	PCRC 2	PCRC 3	PCRC 4	PCRC 5	PCRC 6	PCRC 7	PCRC 8
OTU01	*Streptococcus*	1815	4536	1	1	1	1	3	2	2	2	3	0	1	0
OTU02	*Staphylococcus*	3	0	24	0	0	0	0	1	0	0	0	2	2	3
OTU03	*Bifidobacterium*	4	3	4677	2032	22	2	0	1	0	0	0	0	1	0
OTU04	*Bifidobacterium*	3	0	1047	208	1	0	2	0	0	0	0	1	0	2
OTU05	*Streptococcus*	2140	470	64	3	2	0	0	0	0	0	2	0	0	1
OTU06	*Escherichia-Shigella*	2	0	623	44	217	87	0	0	0	1	1	0	1	1
OTU07	*Gemella*	1925	508	2	1	0	0	1	0	2	1	0	1	0	0
OTU09	*Acinetobacter*	165	716	0	1	0	0	0	0	0	0	0	0	1	0
OTU10	*Bifidobacterium*	0	0	22	1	6	1	0	0	1	0	0	0	1	0
OTU11	*Haemophilus*	232	333	0	0	0	0	0	0	0	0	0	0	0	0
OTU13	*Raoultella*	0	0	947	10	0	0	0	0	0	0	0	0	0	0
OTU16	*Streptococcus*	7	87	4	1	0	0	0	0	0	0	0	0	0	0
OTU18	*Veillonella*	22	2	0	0	0	0	0	0	0	0	0	0	0	0
OTU19	*Rothia*	1347	147	0	0	0	0	0	0	0	0	0	0	0	0
OTU20	*Bacteroides*	0	1	53	9	8	1	0	1	0	0	0	0	4	0
OTU23	*Bacteroides*	1	14	4	30	836	210	0	3	1	0	1	0	102	0
OTU24	*Bifidobacterium*	0	0	104	5	5	0	0	1	0	0	3	0	1	0
OTU25	*Parabacteroides*	1	1	1087	135	8	3	0	0	0	0	0	0	21	0
OTU26	*Dolosigranulum*	2	2	0	0	0	1	0	5704	0	0	0	0	0	0
OTU27	*Akkermansia*	2	0	0	5	58	13	0	0	0	0	2	0	417	0
OTU29	*Enterococcus*	0	0	37	4	0	0	0	0	0	0	0	0	0	0
OTU30	Bacilli_c;RF39_o;RF39_fa;RF39_ge	0	0	0	0	0	0	0	0	0	0	2	0	8991	4
OTU31	*Bifidobacterium*	1	0	343	29	0	0	0	0	0	0	0	0	0	0
OTU33	*Phascolarctobacterium*	1	1	53	27	27	4	0	0	0	0	0	0	536	0
OTU34	*Corynebacterium*	15	0	4	0	0	0	7289	856	2	0	0	0	0	0
OTU35	Bacilli_c;RF39_o;RF39_fa;RF39_ge	1	0	0	0	0	0	0	0	0	0	3	0	2408	0
OTU41	*Bacteroides*	1	3	1	4	92	30	0	0	0	0	0	0	6	0
OTU43	*Faecalibacterium*	1	5	0	36	97	33	0	0	1	0	0	0	23	0
OTU45	*Lactobacillus*	124	123	0	1	0	0	0	0	0	0	0	0	0	0
OTU48	*Streptococcus*	128	53	0	0	0	0	0	0	0	0	0	0	0	0
OTU49	*Streptococcus*	108	52	1	0	0	0	0	0	0	0	0	0	0	0
OTU57	*Lachnospiraceae_ge*	0	0	5	0	4	2	0	0	0	0	0	0	51	0
OTU61	*Bacteroides*	5	1	1	15	181	92	1	2	0	0	0	0	92	0
OTU64	*Bifidobacterium*	2	0	0	0	26	7	2	0	0	0	0	0	2	0
OTU68	*Streptococcus*	48	21	0	0	0	0	0	0	0	0	0	0	0	0
OTU81	*Streptococcus*	33	5	0	0	0	0	0	0	0	0	0	0	0	0
OTU85	*Streptococcus*	58	20	1	0	0	0	0	0	0	0	0	0	0	0
OTU90	*Gemella*	20	15	0	0	0	0	0	0	0	0	0	0	0	0
OTU97	*Streptococcus*	67	44	0	0	0	0	0	0	0	0	0	0	0	0
OTU99	*Bifidobacterium*	0	0	24	0	2	1	0	0	0	0	0	0	0	0
OTU109	*Blautia*	0	0	0	0	40	9	1	0	0	1	0	0	0	0
OTU112	*Anaerostipes*	0	0	0	5	29	8	2	0	0	0	0	0	2	0
OTU114	*Fusicatenibacter*	11	1	0	5	41	10	0	0	0	0	0	0	3	0
OTU118	*Erysipelotrichaceae_UCG-003*	0	0	0	1	29	16	0	0	0	0	0	0	7	0
OTU124	*Bifidobacterium*	0	0	32	4	2	0	0	0	0	0	0	0	0	0
OTU125	*Streptococcus*	37	13	0	0	0	0	0	0	0	0	0	0	0	0
OTU139	*Prevotella*	0	43	0	1	0	0	0	0	0	0	0	0	0	0
OTU155	*Streptococcus*	18	23	0	0	0	0	0	0	0	0	0	0	0	0
OTU156	*Streptococcus*	27	8	0	0	0	0	0	0	0	0	0	0	0	0
OTU167	*Agathobacter*	0	0	0	2	23	21	0	0	0	0	1	0	296	0
OTU168	*Enterobacteriaceae_unclassified*	0	0	52	3	0	0	0	0	0	0	0	0	0	0
OTU170	*Bacteroides*	0	0	0	2	62	20	0	0	0	0	0	0	24	0
OTU192	*Ruminococcus*	0	0	1	0	86	34	0	0	0	0	0	0	0	0
OTU195	*Ruminococcus*	0	0	0	1	2	6	0	0	0	0	0	0	27	0
OTU203	*Bacteroides*	0	1	0	0	38	31	0	0	0	0	0	0	0	0
OTU205	*Bifidobacterium*	1	0	165	15	0	0	0	0	0	0	0	0	0	0
OTU206	*Bacteroides*	1	3	0	0	26	3	0	0	0	0	0	0	2	0
OTU207	*Subdoligranulum*	0	1	0	2	21	8	0	0	0	0	0	0	1	0
OTU213	*Parasutterella*	0	3	0	0	112	29	0	0	0	0	0	0	0	0
OTU217	*Gemella*	21	0	0	0	0	0	0	0	0	0	0	0	0	0
OTU226	*Prevotella*	0	112	0	0	0	0	0	0	0	0	0	0	0	0
OTU254	*Parabacteroides*	0	0	20	0	0	0	0	0	0	0	0	0	0	0
OTU262	*Enterobacteriaceae_unclassified*	0	0	28	2	0	0	0	0	0	0	0	0	0	0
OTU272	*Bifidobacterium*	0	1	70	16	0	0	0	0	0	0	0	0	0	0
OTU276	*Lachnospiraceae_ge*	1	0	1	4	21	9	0	0	0	0	0	0	2	0
OTU285	*Enterobacteriaceae_unclassified*	0	0	28	2	0	0	0	0	0	0	0	0	0	0
OTU292	*Bifidobacterium*	0	0	48	20	0	0	0	0	0	0	0	0	0	0
OTU300	*Bifidobacterium*	0	0	66	17	0	0	0	0	0	0	0	0	0	0
OTU303	*Bifidobacterium*	0	0	33	4	0	0	0	0	0	0	0	0	0	0
OTU306	*Bifidobacterium*	0	0	38	3	0	0	0	0	0	0	0	0	0	0
OTU318	*Enterobacteriaceae_unclassified*	0	0	30	0	0	0	0	0	0	0	0	0	0	0
OTU322	*Enterobacteriaceae_unclassified*	0	0	22	0	0	0	0	0	0	0	0	0	0	0
OTU357	*Acinetobacter*	2	21	0	0	0	0	0	0	0	0	0	0	0	0
OTU367	*Bifidobacterium*	0	0	41	4	0	0	0	0	0	0	0	0	0	0
OTU380	*Bifidobacterium*	0	0	29	7	0	0	0	0	0	0	0	0	0	0
OTU462	*Campylobacter*	0	162	0	0	0	0	0	0	0	0	0	0	0	0
OTU558	Bacilli_c;RF39_o;RF39_fa;RF39_ge	0	0	0	0	0	0	0	0	0	0	0	0	126	0
OTU624	Bacilli_c;RF39_o;RF39_fa;RF39_ge	0	0	0	0	0	0	0	0	0	0	0	0	111	0
OTU694	*Faecalibacterium*	0	0	0	0	24	3	0	0	0	0	0	0	0	0
OTU726	Bacilli_c;RF39_o;RF39_fa;RF39_ge	0	0	0	0	0	0	0	0	0	0	0	0	84	0
OTU795	*Bacteroides*	0	0	0	0	32	11	0	0	0	0	0	0	0	0
OTU942	Bacilli_c;RF39_o;RF39_fa;RF39_ge	0	0	0	0	0	0	0	0	0	0	0	0	64	0
OTU1372	Bacilli_c;RF39_o;RF39_fa;RF39_ge	0	0	0	0	0	0	0	0	0	0	0	0	37	0
OTU1827	Bacilli_c;RF39_o;RF39_fa;RF39_ge	0	0	0	0	0	0	0	0	0	0	0	0	28	0
OTU2087	Bacilli_c;RF39_o;RF39_fa;RF39_ge	0	0	0	0	0	0	0	0	0	0	0	0	26	0
Others	Others	3283	2255	3614	728	4727	1681	80	59	15	5	20	7	2763	5

“Others” represents OTUs (operational taxonomic unit) accounting for ≤20 reads in negative controls. EC—extraction control; PCRC—polymerase chain reaction control.

**Table A3:** The proportion of mothers and infants at each time point for which an OTU was present.

OTUs	Category	Day 2–5	Day 30	Day 60	Day 90	Day 120
		Maternal Faecal				
OTU01 (*Streptococcus mitis*)	Absent	9 (100%)	9 (100%)	5 (55.56%)	7 (70%)	8 (80%)
	Low	0 (0%)	0 (0%)	3 (33.33%)	3 (30%)	2 (20%)
	High	0 (0%)	0 (0%)	1 (11.11%)	0 (0%)	0 (0%)
OTU23 (*Phocaeicola vulgatus*)	Absent	2 (22.22%)	0 (0%)	1 (11.11%)	1 (10%)	2 (20%)
	Low	5 (55.56%)	3 (33.33%)	3 (33.33%)	5 (50%)	5 (50%)
	Medium	2 (22.22%)	6 (66.67%)	5 (55.56%)	4 (40%)	3 (30%)
OTU43 (*Faecalibacterium prausnitzii*)	Absent	4 (44.44%)	0 (0%)	1 (11.11%)	3 (30%)	1 (10%)
	Low	5 (55.56%)	6 (66.67%)	5 (55.56%)	6 (60%)	8 (80%)
	Medium	0 (0%)	3 (33.33%)	3 (33.33%)	1 (10%)	1 (10%)
OTU64 (*Bifidobacterium adolescentis*)	Absent	5 (55.56%)	5 (55.56%)	6 (66.67%)	4 (40%)	8 (80%)
	Low	3 (33.33%)	3 (33.33%)	3 (33.33%)	4 (40%)	1 (10%)
	Medium	1 (11.11%)	1 (11.11%)	0 (0%)	2 (20%)	1 (10%)
OTU82 (*Oscillibacter* sp.)	Absent	6 (66.67%)	7 (77.78%)	6 (66.67%)	10 (100%)	6 (60%)
	Low	2 (22.22%)	2 (22.22%)	3 (33.33%)	0 (0%)	3 (30%)
	Medium	1 (11.11%)	0 (0%)	0 (0%)	0 (0%)	1 (10%)
OTU107 (*Romboutsia timonensis*)	Absent	7 (77.78%)	5 (55.56%)	5 (55.56%)	2 (20%)	5 (50%)
	Low	1 (11.11%)	4 (44.44%)	4 (44.44%)	7 (70%)	5 (50%)
	Medium	1 (11.11%)	0 (0%)	0 (0%)	1 (10%)	0 (0%)
		**Human Milk**				
OTU02 (*Staphylococcus epidermidis*)	Absent	1 (11.11%)	0 (0%)	2 (20%)	1 (10%)	2 (20%)
	Low	3 (33.33%)	1 (10%)	5 (50%)	4 (40%)	2 (20%)
	Medium	2 (22.22%)	3 (30%)	0 (0%)	3 (30%)	5 (50%)
	High	3 (33.33%)	6 (60%)	3 (30%)	2 (20%)	1 (10%)
OTU05 (*Streptococcus salivarius*)	Absent	1 (11.11%)	3 (30%)	1 (10%)	1 (10%)	1 (10%)
	Low	5 (55.56%)	5 (50%)	3 (30%)	4 (40%)	6 (60%)
	Medium	2 (22.22%)	2 (20%)	3 (30%)	3 (30%)	2 (20%)
	High	1 (11.11%)	0 (0%)	3 (30%)	2 (20%)	1 (10%)
OTU09 (*Acinetobacter johnsonii*)	Absent	9 (100%)	0 (0%)	4 (40%)	0 (0%)	10 (100%)
	Low	0 (0%)	0 (0%)	4 (40%)	0 (0%)	0 (0%)
	Medium	0 (0%)	0 (0%)	1 (10%)	0 (0%)	0 (0%)
	High	0 (0%)	0 (0%)	1 (10%)	0 (0%)	0 (0%)
OTU14 (*Veillonella nakazawae*)	Absent	9 (100%)	6 (60%)	7 (70%)	8 (80%)	5 (50%)
	Low	0 (0%)	4 (40%)	3 (30%)	2 (20%)	4 (40%)
	Medium	0 (0%)	0 (0%)	0 (0%)	0 (0%)	1 (10%)
OTU16 (*Streptococcus lactarius*)	Absent	7 (77.78%)	8 (80%)	6 (60%)	7 (70%)	5 (50%)
	Low	2 (22.22%)	1 (10%)	3 (30%)	1 (10%)	2 (20%)
	Medium	0 (0%)	1 (10%)	1 (10%)	2 (20%)	3 (30%)
OTU26 (*Dolosigranulum pigrum*)	Absent	7 (77.78%)	9 (90%)	10 (100%)	7 (70%)	6 (60%)
	Low	2 (22.22%)	1 (10%)	0 (0%)	1 (10%)	4 (40%)
	Medium	0 (0%)	0 (0%)	0 (0%)	2 (20%)	0 (0%)
OTU28 (*Staphyloccocus hominis*)	Absent	5 (55.56%)	9 (90%)	6 (60%)	8 (80%)	5 (50%)
	Low	3 (33.33%)	1 (10%)	4 (40%)	2 (20%)	5 (50%)
	Medium	1 (11.11%)	0 (0%)	0 (0%)	0 (0%)	0 (0%)
		**Infant Oral**				
OTU01 (*Streptococcus mitis*)	Absent	1 (10%)	0 (0%)	0 (0%)	0 (0%)	0 (0%)
	Low	3 (30%)	4 (40%)	4 (40%)	1 (10%)	1 (10%)
	Medium	6 (60%)	4 (40%)	2 (20%)	2 (20%)	3 (30%)
	High	0 (0%)	2 (20%)	4 (40%)	7 (70%)	6 (60%)
OTU02 (*Staphylococcus epidermidis*)	Absent	4 (40%)	5 (50%)	8 (80%)	9 (90%)	10 (100%)
	Low	2 (20%)	5 (50%)	2 (20%)	1 (10%)	0 (0%)
	Medium	3 (30%)	0 (0%)	0 (0%)	0 (0%)	0 (0%)
	High	1 (10%)	0 (0%)	0 (0%)	0 (0%)	0 (0%)
OTU05 (*Streptococcus salivarius*)	Absent	5 (50%)	2 (20%)	4 (40%)	4 (40%)	8 (80%)
	Low	3 (30%)	7 (70%)	4 (40%)	5 (50%)	1 (10%)
	Medium	1 (10%)	0 (0%)	1 (10%)	1 (10%)	1 (10%)
	High	1 (10%)	1 (10%)	1 (10%)	0 (0%)	0 (0%)
OTU11 (*Haemophilus haemolyticus*)	Absent	2 (20%)	6 (60%)	6 (60%)	7 (70%)	2 (20%)
	Low	7 (70%)	2 (20%)	1 (10%)	1 (10%)	4 (40%)
	Medium	0 (0%)	1 (10%)	3 (30%)	2 (20%)	3 (30%)
	High	1 (10%)	1 (10%)	0 (0%)	0 (0%)	1 (10%)
OTU14 (*Veillonella nakazawae*)	Absent	9 (90%)	5 (50%)	7 (70%)	6 (60%)	2 (20%)
	Low	1 (10%)	2 (20%)	2 (20%)	3 (30%)	8 (80%)
	Medium	0 (0%)	2 (20%)	1 (10%)	1 (10%)	0 (0%)
	High	0 (0%)	1 (10%)	0 (0%)	0 (0%)	0 (0%)
OTU16 (*Streptococcus lactarius*)	Absent	8 (80%)	9 (90%)	6 (60%)	6 (60%)	3 (30%)
	Low	2 (20%)	1 (10%)	4 (40%)	3 (30%)	6 (60%)
	Medium	0 (0%)	0 (0%)	0 (0%)	1 (10%)	1 (10%)
OTU28 (*Staphyloccocus hominis*)	Absent	5 (50%)	8 (80%)	9 (90%)	10 (100%)	10 (100%)
	Low	4 (40%)	2 (20%)	1 (10%)	0 (0%)	0 (0%)
	Medium	1 (10%)	0 (0%)	0 (0%)	0 (0%)	0 (0%)
		**Infant Faecal**				
OTU02 (*Staphylococcus epidermidis*)	Absent	2 (20%)	3 (30%)	3 (30%)	6 (60%)	7 (70%)
	Low	5 (50%)	6 (60%)	6 (60%)	4 (40%)	3 (30%)
	Medium	3 (30%)	1 (10%)	1 (10%)	0 (0%)	0 (0%)
OTU05 (*Streptococcus salivarius*)	Absent	3 (30%)	1 (10%)	5 (50%)	5 (50%)	7 (70%)
	Low	4 (40%)	7 (70%)	4 (40%)	5 (50%)	3 (30%)
	Medium	3 (30%)	1 (10%)	1 (10%)	0 (0%)	0 (0%)
	High	0 (0%)	1 (10%)	0 (0%)	0 (0%)	0 (0%)
OTU14 (*Veillonella nakazawae*)	Absent	9 (90%)	10 (100%)	5 (50%)	8 (80%)	6 (60%)
	Low	0 (0%)	0 (0%)	5 (50%)	1 (10%)	2 (20%)
	Medium	1 (10%)	0 (0%)	0 (0%)	1 (10%)	2 (20%)
OTU20 (*Bacteroides fragilis*)	Absent	10 (100%)	8 (80%)	4 (40%)	10 (100%)	8 (80%)
	Low	0 (0%)	2 (20%)	5 (50%)	0 (0%)	1 (10%)
	Medium	0 (0%)	0 (0%)	0 (0%)	0 (0%)	1 (10%)
	High	0 (0%)	0 (0%)	1 (10%)	0 (0%)	0 (0%)
OTU24 (*Bifidobacterium longum*)	Absent	3 (30%)	7 (70%)	4 (40%)	6 (60%)	7 (70%)
	Low	3 (30%)	1 (10%)	4 (40%)	4 (40%)	2 (20%)
	Medium	4 (40%)	2 (20%)	2 (20%)	0 (0%)	1 (10%)

Results are presented as the number of participants having OTUs (operational taxonomic unit) (percentage of participants).

**Table A4:** Associations between individual HMO intake and infant oral bacterial composition.

Bacterial OTUs	Comparison (Parameter—Intercept)	Estimated Difference	SE	*p*-Value
		Day 30		
		LNnT		
OTU11 (*Haemophilus haemolyticus*)	Low—Absent	83.375	30.368	0.034
OTU11 (*Haemophilus haemolyticus*)	High—Absent	240.073	40.174	0.001
		LNFP I		
OTU18 (*Veillonella* sp. oral clone ASCB03)	High—Absent	1541.626	351.386	0.003
OTU32 (*Haemophilus parainfluenzae*)	Low—Absent	1502.522	332.410	0.003
		LNFP III		
OTU18 (*Veillonella* sp. oral clone ASCB03)	High—Absent	44.042	3.996	<0.001
OTU32 (*Haemophilus parainfluenzae*)	Low—Absent	44.201	3.754	<0.001
		LSTc		
OTU18 (*Veillonella* sp. oral clone ASCB03)	High—Absent	233.322	40.045	<0.001
OTU32 (*Haemophilus parainfluenzae*)	Low—Absent	245.268	31.792	<0.001
		LNH		
OTU11 (*Haemophilus haemolyticus*)	High—Absent	64.315	17.900	0.012
		DSLNT		
OTU11 (*Haemophilus haemolyticus*)	Low—Absent	113.917	28.901	0.008
OTU18 (*Veillonella* sp. oral clone ASCB03)	Low—Absent	−51.026	20.311	0.040
OTU18 (*Veillonella* sp. oral clone ASCB03)	High—Absent	110.395	33.168	0.013
OTU32 (*Haemophilus parainfluenzae*)	Low—Absent	124.808	34.216	0.008
		FLNH		
OTU05 (*Streptococcus salivarius*)	High—Absent	323.804	94.591	0.011
		**Day 60**		
		3FL		
OTU02 (*Staphylococcus epidermidis*)	Low—Absent	−550.146	203.242	0.027
		DFLac		
OTU32 (*Haemophilus parainfluenzae*)	Medium—Absent	224.329	70.758	0.016
		LNnT		
OTU03 (*Bifidobacterium longum* subsp. *infantis*)	Low—Absent	100.502	31.407	0.015
		LNFP I		
OTU18 (*Veillonella* sp. oral clone ASCB03)	Medium—Absent	788.647	204.195	0.006
		LNFP II		
OTU02 (*Staphylococcus epidermidis*)	Low—Absent	−381.626	155.937	0.040
		LNFP III		
OTU18 (*Veillonella* sp. oral clone ASCB03)	Medium—Absent	11.980	3.171	0.007
		LSTb		
OTU14 (*Veillonella nakazawae*)	Low—Absent	42.776	15.187	0.026
		LSTc		
OTU01 (*Streptococcus mitis*)	Medium—Low	69.136	17.701	0.006
		DFLNT		
OTU14 (*Veillonella nakazawae*)	Low—Absent	553.393	176.628	0.017
		DSLNT		
OTU14 (*Veillonella nakazawae*)	Low—Absent	70.404	20.949	0.012
		**Day 90**		
		DFLac		
OTU01 (*Streptococcus mitis*)	Medium—Low	326.879	98.311	0.013
OTU32 (*Haemophilus parainfluenzae*)	Medium—Absent	321.837	79.181	0.004
		3′SL		
OTU01 (*Streptococcus mitis*)	Medium—Low	115.877	48.265	0.047
OTU32 (*Haemophilus parainfluenzae*)	Medium—Absent	167.484	20.209	<0.001
		6′SL		
OTU19 (*Rothia mucilaginosa*)	Low—Absent	−60.645	18.879	0.015
OTU19 (*Rothia mucilaginosa*)	Medium—Absent	−85.766	30.830	0.027
		LNT		
OTU05 (*Streptococcus salivarius*)	Low—Absent	−275.077	47.665	<0.001
		LNFP I		
OTU18 (*Veillonella* sp. oral clone ASCB03)	Medium—Absent	242.589	62.818	0.008
		LNFP II		
OTU22 (*Bergeyella* sp.)	Low—Absent	−251.985	90.486	0.024
		LSTb		
OTU07 (*Gemella haemolysans*)	Low—Absent	−39.158	10.458	0.007
OTU07 (*Gemella haemolysans*)	Medium—Absent	−28.050	9.783	0.024
		LSTc		
OTU32 (*Haemophilus parainfluenzae*)	Medium—Absent	68.280	18.015	0.005
		DSLNT		
OTU07 (*Gemella haemolysans*)	Low—Absent	−56.769	14.608	0.006
OTU07 (*Gemella haemolysans*)	Medium—Absent	−44.494	13.664	0.014
OTU18 (*Veillonella* sp. oral clone ASCB03)	Medium—Absent	45.434	9.703	0.003
		DSLNH		
OTU19 (*Rothia mucilaginosa*)	Low—Absent	−46.494	11.472	0.005
		**Day 120**		
		2′FL		
OTU03 (*Bifidobacterium longum* subsp. *infantis*)	Low—Absent	1044.095	324.676	0.012
OTU18 (*Veillonella* sp. oral clone ASCB03)	Medium—Absent	1143.659	348.875	0.014
		3FL		
OTU03 (*Bifidobacterium longum* subsp. *infantis*)	Low—Absent	−597.110	241.857	0.039
OTU18 (*Veillonella* sp. oral clone ASCB03)	Medium—Absent	−736.135	221.592	0.013
		3′SL		
OTU32 (*Haemophilus parainfluenzae*)	Low—Absent	112.458	11.639	<0.001
		6′SL		
OTU01 (*Streptococcus mitis*)	High—Low	−58.021	20.220	0.024
OTU03 (*Bifidobacterium longum* subsp. *infantis*)	Low—Absent	63.180	18.410	0.009
OTU18 (*Veillonella* sp. oral clone ASCB03)	Medium—Absent	67.677	20.171	0.012
		LNFP I		
OTU03 (*Bifidobacterium longum* subsp. *infantis*)	Low—Absent	291.768	88.867	0.011
OTU18 (*Veillonella* sp. oral clone ASCB03)	Medium—Absent	323.706	93.534	0.011
		LNH		
OTU03 (*Bifidobacterium longum* subsp. *infantis*)	Low—Absent	31.952	9.363	0.009
OTU18 (*Veillonella* sp. oral clone ASCB03)	Medium—Absent	32.123	10.591	0.019
		DSLNT		
OTU01 (*Streptococcus mitis*)	High—Low	−58.388	21.383	0.029
		FDSLNH		
OTU05 (*Streptococcus salivarius*),	Low—Absent	307.528	96.080	0.015
		DSLNH		
OTU01 (*Streptococcus mitis*)	High—Low	−22.305	8.279	0.031
OTU03 (*Bifidobacterium longum* subsp. infantis)	Low—Absent	32.307	11.696	0.025

The OTUs (operational taxonomic unit) forming >1% of relative abundance in infant oral samples were only included in the ANOVA analysis and the OTU relative abundance was categorized into four levels as high = >30%, medium = 5–30%, low = <5% and absent. Estimated difference represents HMO difference from the reference group. 2′FL—2′-fucosyllactose; 3′SL—3′-sialyllactose; 3FL—3-fucosyllactose; 6′SL—6′-sialyllactose; DFLac—difucosyllactose; DFLNH—difucosyllacto-N-hexaose; DFLNT—difucosyllacto-N-tetrose; DSLNH—disialyllacto-N-hexaose; DSLNT—disialyllacto-N-tetraose; FDSLNH—fucodisialyllacto-N-hexaose; FLNH—fucosyllacto-N-hexaose; LNFP I—lacto-N-fucopentaose; LNFP II—lacto-N-fucopentaose II; LNFP III—lacto-N-fucopentaose; LNH—lacto-N-hexaose; LNnT—lacto-N-neotetraose; LNT—lacto-N-tetrose; LSTb—sialyl-lacto-N-tetraose b; LSTc—sialyl-lacto-N-tetraose c.

**Table A5:** Associations between individual HMO intake and infant faecal bacterial composition.

Bacterial OTUs	Comparison (Parameter—Intercept)	Estimated Difference	SE	*p*-Value
		Day 30		
		2′FL		
OTU04 (*Bifidobacterium breve*)	Low—Absent	−1666.940	425.648	0.008
		3FL		
OTU13 (*Raoultella ornithinolytica*)	High—Absent	735.702	258.385	0.025
OTU24 (*Bifidobacterium longum*)	Low—Absent	723.455	265.973	0.030
		DFLac		
OTU04 (*Bifidobacterium breve*)	Low—Absent	−138.030	28.935	0.003
		**3′SL**		
OTU03 (*Bifidobacterium longum* subsp. *infantis*)	Medium—Absent	−41.128	14.211	0.028
OTU20 (*Bacteroides fragilis*)	Low—Absent	48.548	19.523	0.038
		LNnT		
OTU03 (*Bifidobacterium longum* subsp. *infantis*)	Low—Absent	205.125	55.873	0.010
OTU17 (*Klebsiella pneumoniae*)	Low—Absent	232.141	40.266	<0.001
OTU20 (*Bacteroides fragilis*)	Low—Absent	126.620	53.970	0.047
		LNFP I		
OTU05 (*Streptococcus salivarius*)	Medium—Absent	1813.872	453.887	0.007
OTU10 (*Bifidobacterium pseudocatenulatum*)	High—Absent	1475.480	350.940	0.006
OTU17 (*Klebsiella pneumoniae*)	High—Absent	1492.947	323.246	0.002
OTU25 (*Parabacteroides distasonis*)	Low—Absent	1524.848	316.978	0.001
		LNFP II		
OTU17 (*Klebsiella pneumoniae*)	Low—Absent	484.536	170.968	0.025
		LNFP III		
OTU05 (*Streptococcus salivarius*)	Low—Absent	5.726	2.302	0.047
OTU05 (*Streptococcus salivarius*)	Medium—Absent	50.291	3.045	<0.001
OTU05 (*Streptococcus salivarius*)	High—Absent	11.672	3.045	0.009
OTU10 (*Bifidobacterium pseudocatenulatum*)	High—Absent	44.116	4.077	<0.001
OTU17 (*Klebsiella pneumoniae*)	High—Absent	44.575	3.925	<0.001
OTU25 (*Parabacteroides distasonis*)	Low—Absent	44.540	3.651	<0.001
		LSTb		
OTU24 (*Bifidobacterium longum*)	Low—Absent	49.510	17.349	0.025
		LSTc		
OTU05 (*Streptococcus salivarius*)	Medium—Absent	281.536	46.123	<0.001
OTU10 (*Bifidobacterium pseudocatenulatum*)	High—Absent	247.574	33.782	<0.001
OTU17 (*Klebsiella pneumoniae*)	Low—Absent	70.097	19.648	0.009
OTU17 (*Klebsiella pneumoniae*)	High—Absent	241.556	37.923	<0.001
OTU25 (*Parabacteroides distasonis*)	Low—Absent	238.437	36.608	<0.001
		DFLNT		
OTU04 (*Bifidobacterium breve*)	Low—Absent	−597.364	136.154	0.005
		DSLNT		
OTU10 (*Bifidobacterium pseudocatenulatum*)	High—Absent	124.563	37.241	0.016
OTU25 (*Parabacteroides distasonis*)	Low—Absent	133.074	41.169	0.012
		FLNH		
OTU13 (*Raoultella ornithinolytica*)	High—Absent	342.258	81.920	0.004
OTU24 (*Bifidobacterium longum*)	Low—Absent	351.494	83.120	0.004
		DFLNH		
OTU20 (*Bacteroides fragilis*)	Low—Absent	88.416	37.035	0.044
		DSLNH		
OTU20 (*Bacteroides fragilis*)	Low—Absent	35.677	15.099	0.046
		**Day 60**		
		3FL		
OTU05 (*Streptococcus salivarius*)	Low—Absent	−457.444	140.352	0.014
OTU05 (*Streptococcus salivarius*)	Medium—Absent	−721.382	229.193	0.016
		LNT		
OTU06 (*Escherichia coli*)	Medium—Absent	498.920	145.761	0.014
OTU29 (*Enterococcus faecalis*)	Low—Absent	636.978	249.903	0.034
		LNnT		
OTU17 (*Klebsiella pneumoniae*)	Low—Absent	76.183	23.105	0.013
OTU17 (*Klebsiella pneumoniae*)	Medium—Absent	70.373	23.105	0.019
		LNFP I		
OTU25 (*Parabacteroides distasonis*)	Low—Absent	700.864	210.358	0.013
		LNFP II		
OTU29 (*Enterococcus faecalis*)	Low—Absent	520.197	204.372	0.034
		LNFP III		
OTU25 (*Parabacteroides distasonis*)	Low—Absent	11.677	2.660	0.003
		LSTb		
OTU06 (*Escherichia coli*)	Medium—Absent	39.951	11.096	0.011
OTU25 (*Parabacteroides distasonis*)	Low—Absent	55.080	20.155	0.029
		DFLNT		
OTU25 (*Parabacteroides distasonis*)	Low—Absent	666.649	211.012	0.016
		LNH		
OTU10 (*Bifidobacterium pseudocatenulatum*)	Low—Absent	26.025	6.182	0.006
OTU10 (*Bifidobacterium pseudocatenulatum*)	Medium—Absent	15.904	6.182	0.042
OTU10 (*Bifidobacterium pseudocatenulatum*)	High—Absent	42.040	8.094	0.002
		FLNH		
OTU20 (*Bacteroides fragilis*)	Low—Absent	−145.411	41.110	0.010
OTU20 (*Bacteroides fragilis*)	High—Absent	−191.839	68.517	0.027
OTU29 (*Enterococcus faecalis*)	Low—Absent	194.456	83.620	0.049
		DFLNH		
OTU04 (*Bifidobacterium breve*)	Medium—Absent	66.004	21.207	0.021
OTU04 (*Bifidobacterium breve*)	High—Absent	65.513	16.197	0.007
		**Day 90**		
		6′SL		
OTU24 (*Bifidobacterium longum*)	Low—Absent	−53.183	22.870	0.049
		LNnT		
OTU13 (*Raoultella ornithinolytica*)	Low—Absent	139.726	45.827	0.019
OTU29 (*Enterococcus faecalis*)	Low—Absent	141.980	42.530	0.010
		LNFP III		
OTU25 (*Parabacteroides distasonis*)	Low—Absent	5.314	2.284	0.048
OTU29 (*Enterococcus faecalis*)	Low—Absent	7.553	2.902	0.032
		LSTc		
OTU03 (*Bifidobacterium longum* subsp. *infantis*)	Low—Absent	56.415	14.472	0.008
		DFLNT		
OTU24 (*Bifidobacterium longum*)	Low—Absent	−259.858	97.869	0.029
		FLNH		
OTU04 (*Bifidobacterium breve*)	High—Absent	−60.398	20.607	0.019
		FDSLNH		
OTU25 (*Parabacteroides distasonis*)	Low—Absent	187.335	78.648	0.044
		**Day 120**		
		2′FL		
OTU05 (*Streptococcus salivarius*)	Low—Absent	794.461	324.411	0.040
		3FL		
OTU05 (*Streptococcus salivarius*)	Low—Absent	−579.838	191.043	0.016
		DFLac		
OTU24 (*Bifidobacterium longum*)	Low—Absent	−143.516	16.277	<0.001
OTU25 (*Parabacteroides distasonis*)	Low—Absent	−130.102	56.234	0.049
		3′SL		
OTU29 (*Enterococcus faecalis*)	Low—Absent	113.907	12.184	<0.001
		LNT		
OTU06 (*Escherichia coli*)	High—Absent	425.170	137.772	0.022
OTU25 (*Parabacteroides distasonis*)	Low—Absent	402.129	149.964	0.028
		LNFP I		
OTU20 (*Bacteroides fragilis*)	Low—Absent	394.347	125.257	0.016
		LNFP II		
OTU05 (*Streptococcus salivarius*)	Low—Absent	−307.140	122.055	0.036
		LSTb		
OTU06 (*Escherichia coli*)	High—Absent	37.797	14.733	0.043
		DFLNT		
OTU06 (*Escherichia coli*)	Medium—Absent	358.632	107.565	0.016
		LNH		
OTU20 (*Bacteroides fragilis*)	Low—Absent	47.773	7.970	<0.001
		FLNH		
OTU06 (*Escherichia coli*)	High—Absent	91.548	35.911	0.044
OTU25 (*Parabacteroides distasonis*)	Low—Absent	105.044	39.804	0.030
		DFLNH		
OTU14 (*Veillonella nakazawae*)	Low—Absent	−43.460	16.298	0.032
		FDSLNH		
OTU24 (*Bifidobacterium longum*)	Low—Absent	226.449	71.658	0.016
OTU25 (*Parabacteroides distasonis*)	Low—Absent	317.661	94.893	0.010

The OTUs (operational taxonomic unit) forming >1% of relative abundance in infant oral samples were only included in the ANOVA analysis and the OTU relative abundance was categorized into four levels as high = > 30%, medium = 5–30%, low = < 5% and absent. Estimated difference represent HMO difference from the reference group. 2′FL—2′-fucosyllactose; 3′SL—3′-sialyllactose; 3FL—3-fucosyllactose; 6′SL—6′-sialyllactose; DFLac—difucosyllactose; DFLNH—difucosyllacto-N-hexaose; DFLNT—difucosyllacto-N-tetrose; DSLNH—disialyllacto-N-hexaose; DSLNT—disialyllacto-N-tetraose; FDSLNH—fucodisialyllacto-N-hexaose; FLNH—fucosyllacto-N-hexaose; LNFP I—lacto-N-fucopentaose; LNFP II—lacto-N-fucopentaose II; LNFP III—lacto-N-fucopentaose; LNH—lacto-N-hexaose; LNnT—lacto-N-neotetraose; LNT—lacto-N-tetrose; LSTb—sialyl-lacto-N-tetraose b; LSTc—sialyl-lacto-N-tetraose c.
